# Supplementary material for: Structural studies of the IFNλ4 receptor complex using cryoEM enabled by protein engineering
Source: Nat Commun. 2025 Jan 18;16:818. doi: 10.1038/s41467-025-56119-y (PMC11742915; doi:10.1038/s41467-025-56119-y)
Supplement: Supplementary file 1 — Supplementary Information [file 41467_2025_56119_MOESM1_ESM.pdf]

# Supplementary Information

for

**"Structural studies of the IFNλ4 receptor complex using cryoEM enabled by protein engineering"**

Grubbe et. al.

## Supplementary Figures

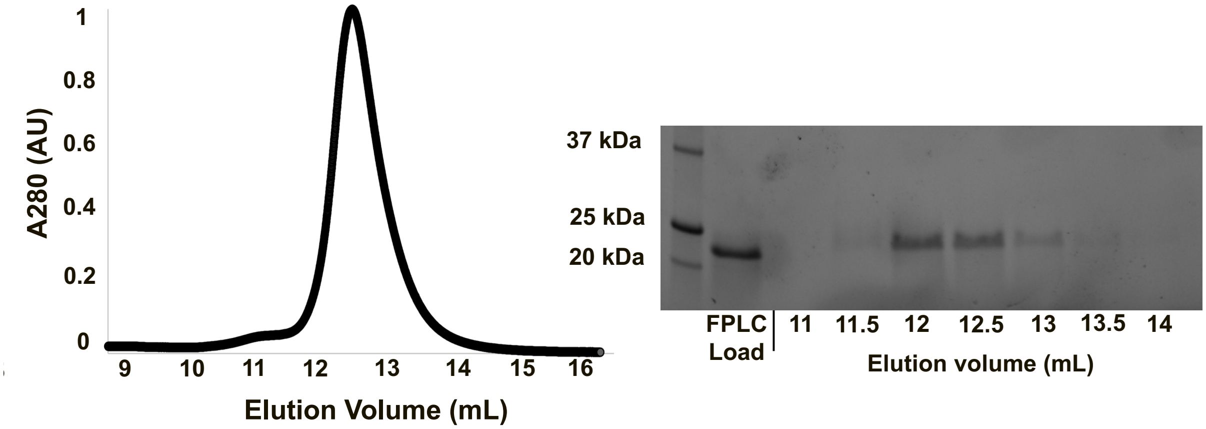


**Supplementary Figure 1: High-yield expression and purification of IFNλ4.** Left: Fast-protein liquid chromatography trace of IFNλ4 protein. Right: SDS-PAGE protein gel of IFNλ4 fractions from accompanying FPLC run.

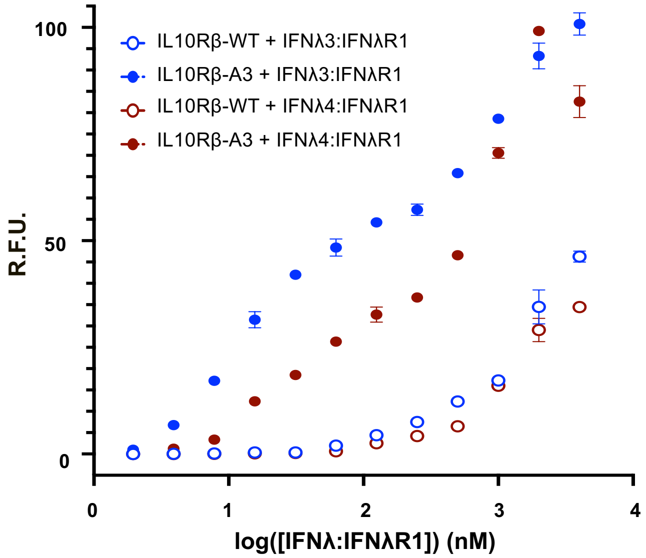


**Supplementary Figure 2: IL10Rβ-A3 greatly improves binding for IFNλ:IFNλR1 measured by yeast display.** Yeast displaying either IL10Rβ (WT) or IL10Rβ-A3 (A3) titrated with equimolar ratios of either IFNλ3 or IFNλ4 with IFNλR1. (n = 2, data plotted with SEM).

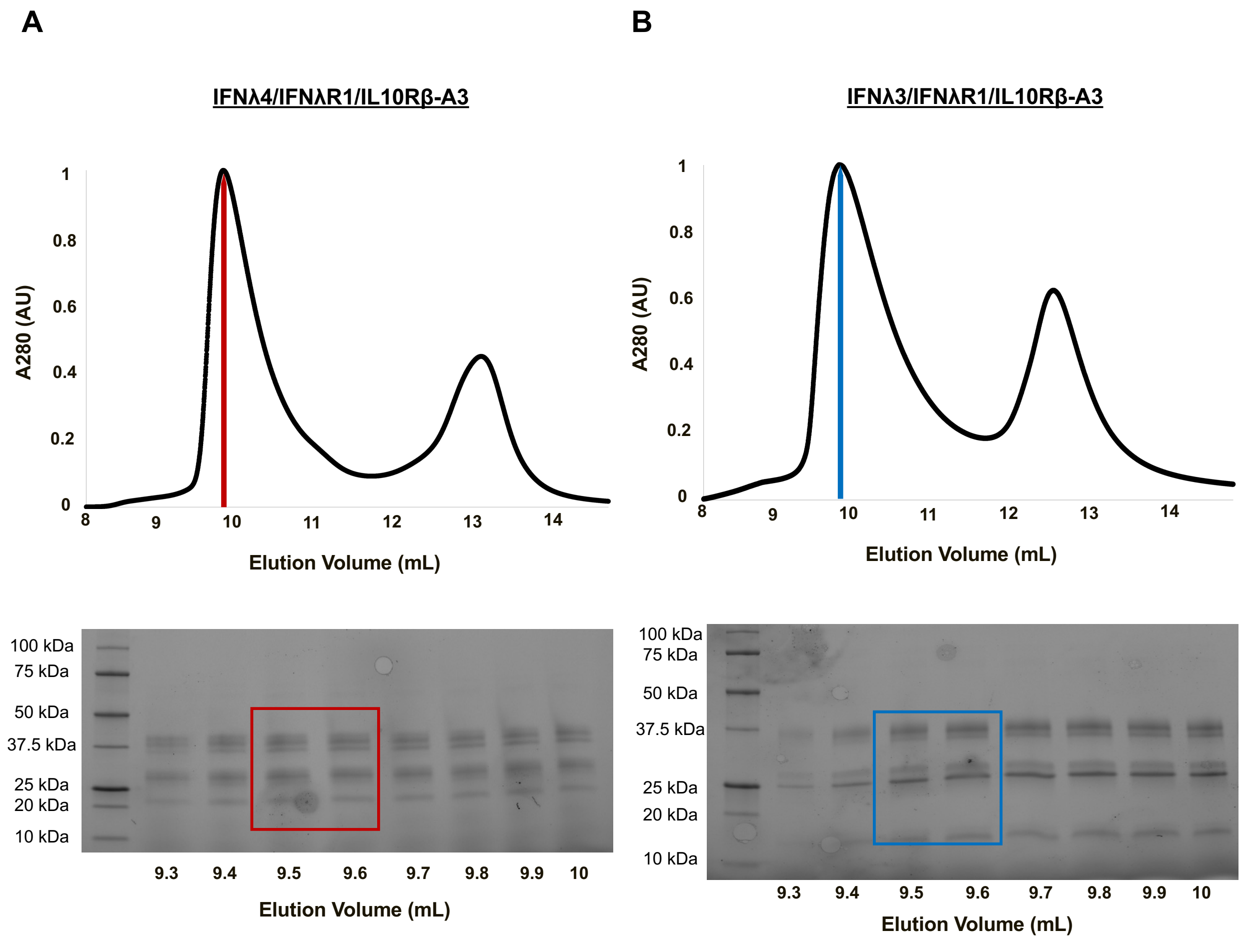


**Supplementary Figure 3: IL10Rβ-A3 enables purification of the ternary complexes formed with IFNλ4:IFNλR1 and IFNλ3:IFNλR1.** A) Size exclusion chromatograms (Superdex S75 column) of the IFNλ4/IFNλR1/IL10Rβ-A3 ternary complex (top). Volume fractions are shown on the SDS-PAGE protein gel (bottom). The red area and square indicate the fractions used for data collection. The peak at ~13 mL is unbound excess IL10Rβ-A3. B) Size exclusion chromatograms of the IFNλ3/IFNλR1/IL10Rβ-A3 ternary complex (top). Volume fractions are shown on the SDS-PAGE protein gel (bottom). The blue area and square indicate the fractions used for data collection. The peak at ~13 mL is unbound excess IL10Rβ-A3.

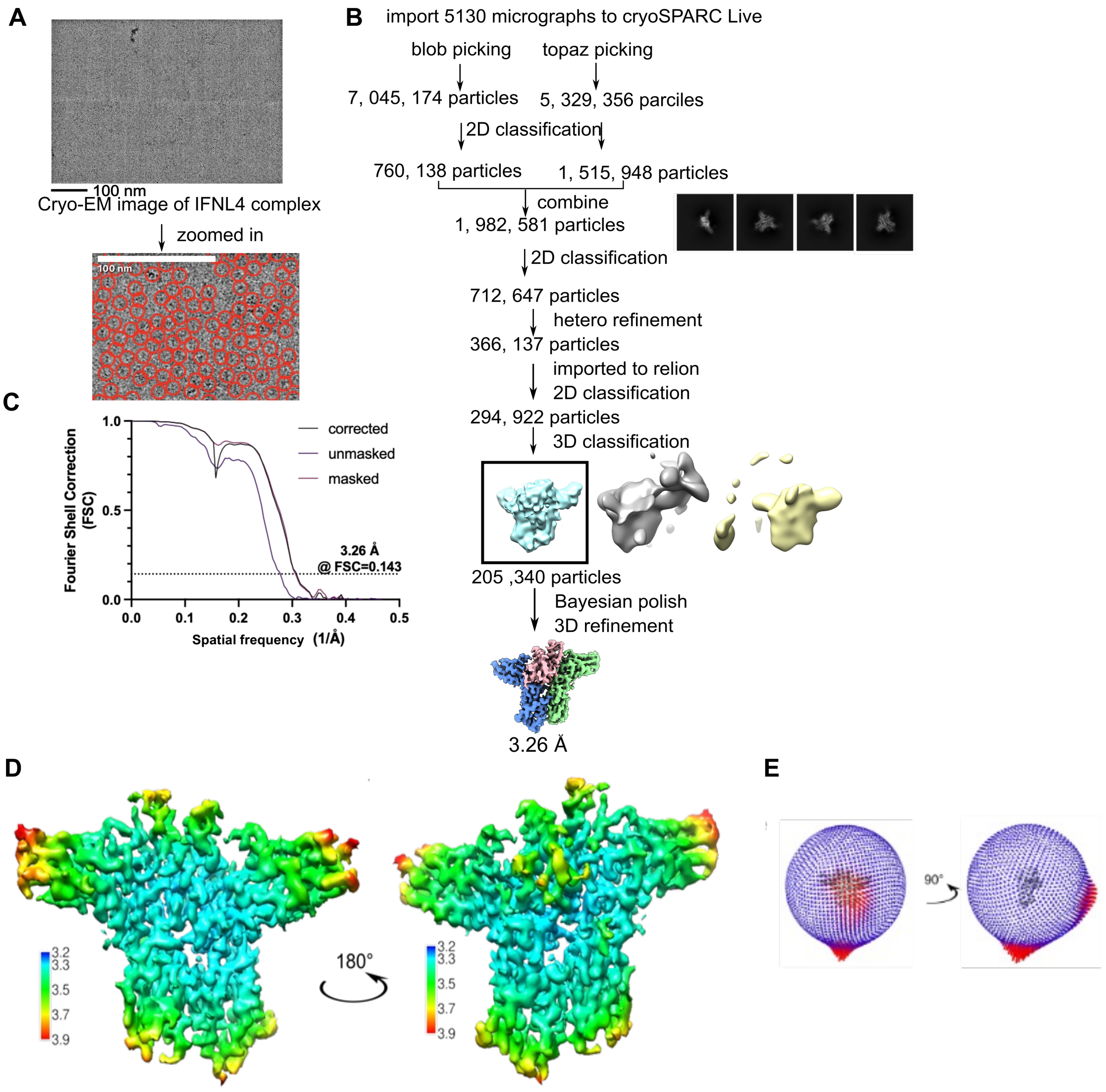


**Supplementary Figure 4: Overview of the workflow, particle picking, and model refinement of the IFNλ4 receptor complex. A)** Image of the IFNλ4 complex on grids. Particles circled in red. **B)** Step-by-step schematic detailing the solution of the IFNλ4 receptor complex to a final resolution of 3.26 Å. (Blue = IL10Rβ-A3, Red = IFNλ4, Green = IFNλR1) **C)** Fourier Shell Correction (FSC) versus spatial frequency for the final model of the IFNλ4 receptor complex. **D)** Local resolution map for the IFNλ4 receptor complex. **E)** Angular distribution of particles used in the final reconstruction.

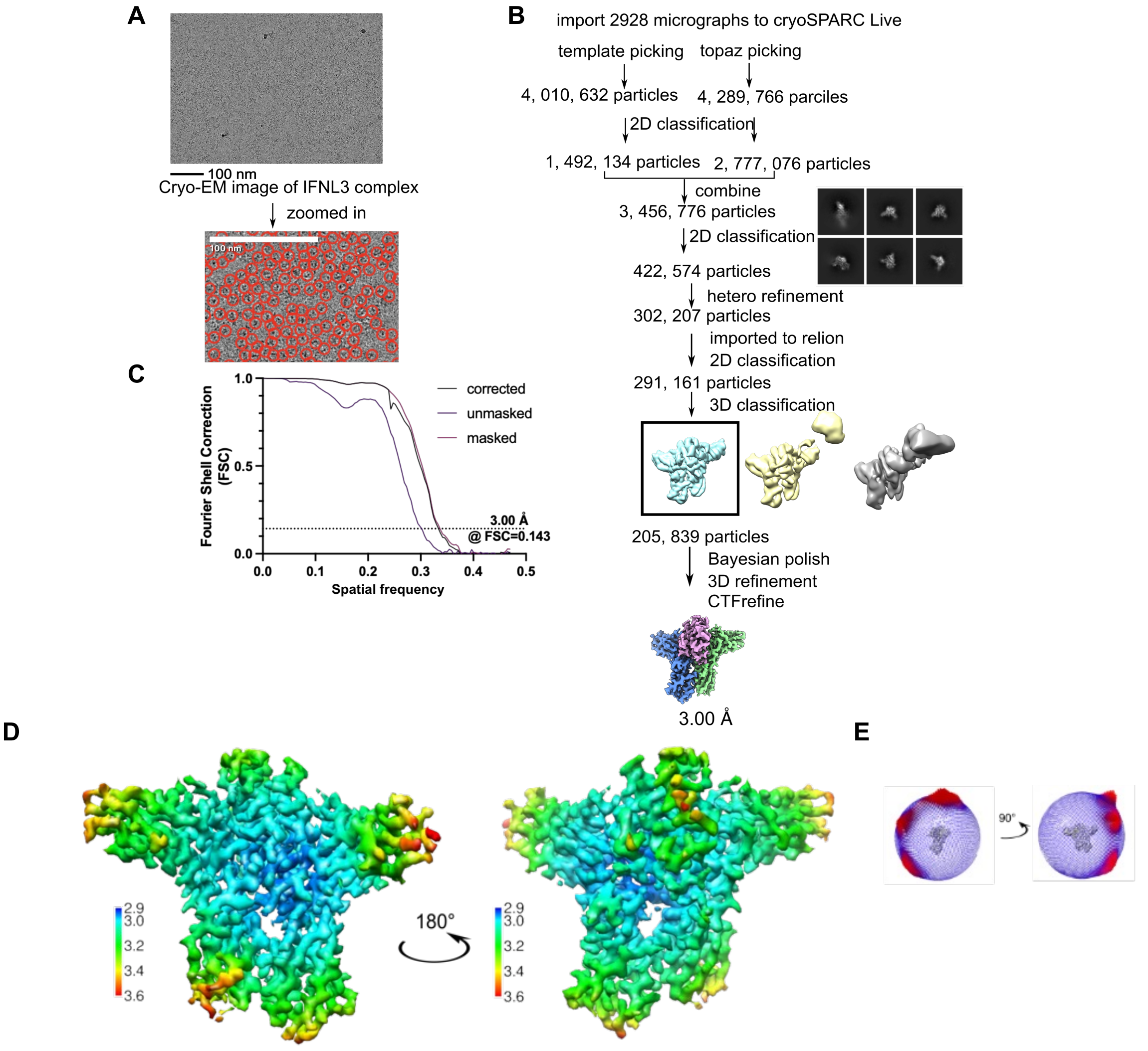


**Supplementary Figure 5: Overview of the workflow, particle picking, and model refinement of the IFNλ3 receptor complex. A)** Image of the IFNλ3 complex on grids. Particles circled in red. **B)** Step-by-step schematic detailing the solution of the IFNλ3 receptor complex to a final resolution of 3.00 Å. (Blue = IL10Rβ-A3, Red = IFNλ3, Green = IFNλR1) **C)** Fourier Shell Correction (FSC) versus spatial frequency for the final model of the IFNλ3 receptor complex. **D)** Local resolution map for the IFNλ3 receptor complex. **E)** Angular distribution of particles used in the final reconstruction.

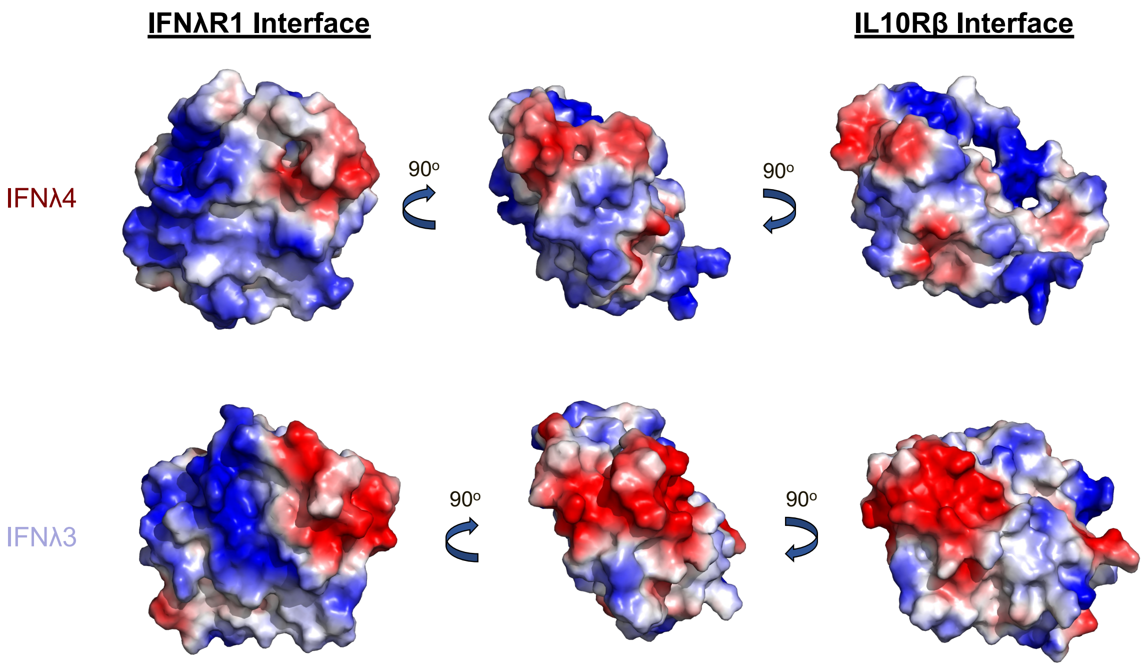


**Supplementary Figure 6: Differences in surface charge for IFNλ4 and IFNλ3.** Differences in surface charge are shown for the IFNλ4 (top row) and IFNλ3 (bottom row) proteins. Blue = basic residue (positive charge), white = uncharged residue, red = acidic residue (negative charge).

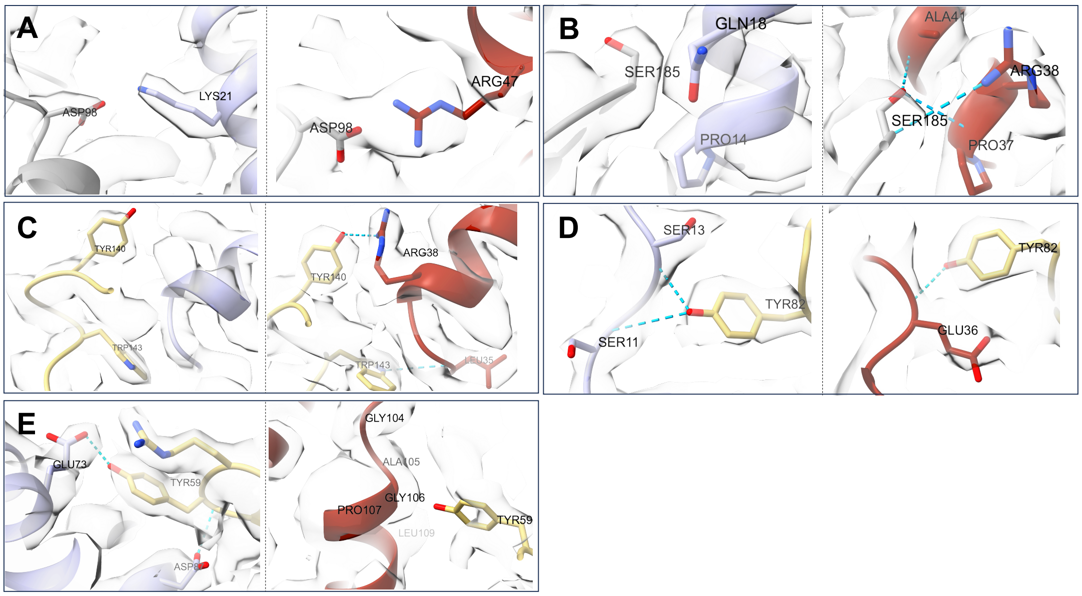


**Supplementary Figure 7: Detailed interactions at IFNλ3 and IFNλ4 ligand/receptor interface. A)** Asp98 on IFNλR1 interaction with Lys21of IFNλ3 and Arg47 of IFNλ4. **B)** Ser185 on IFNλR1 interaction with IFNλ3 and IFNλ4. **C)** Tyr140 and Trp143 on IL10Rβ-A3 interaction with IFNλ3 and IFNλ4. **D)** Tyr82 on IL10Rβ-A3 interaction with IFNλ3 and IFNλ4. **E)** Tyr59 on IL10Rβ-A3 interaction with IFNλ3 and IFNλ4. Left panel: IFNλ3, right panel: IFNλ4, IFNλR1 (gray), IL10Rβ-A3 (gold), IFNλ3 (blue), IFNλ4 (red).

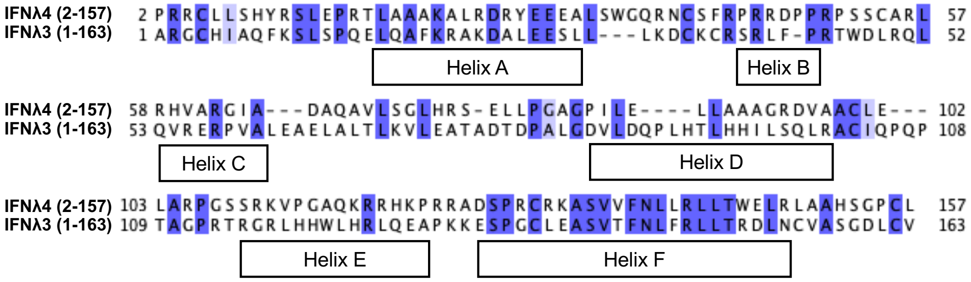


**Supplementary Figure 8: Amino acid sequence alignment of IFNλ4 and IFNλ3.** Helix labels are placed underneath the corresponding amino acid sequences. The residues highlighted in dark blue are identical. Residues highlighted in light blue are highly similar. Residues are numbered in true numerical order.

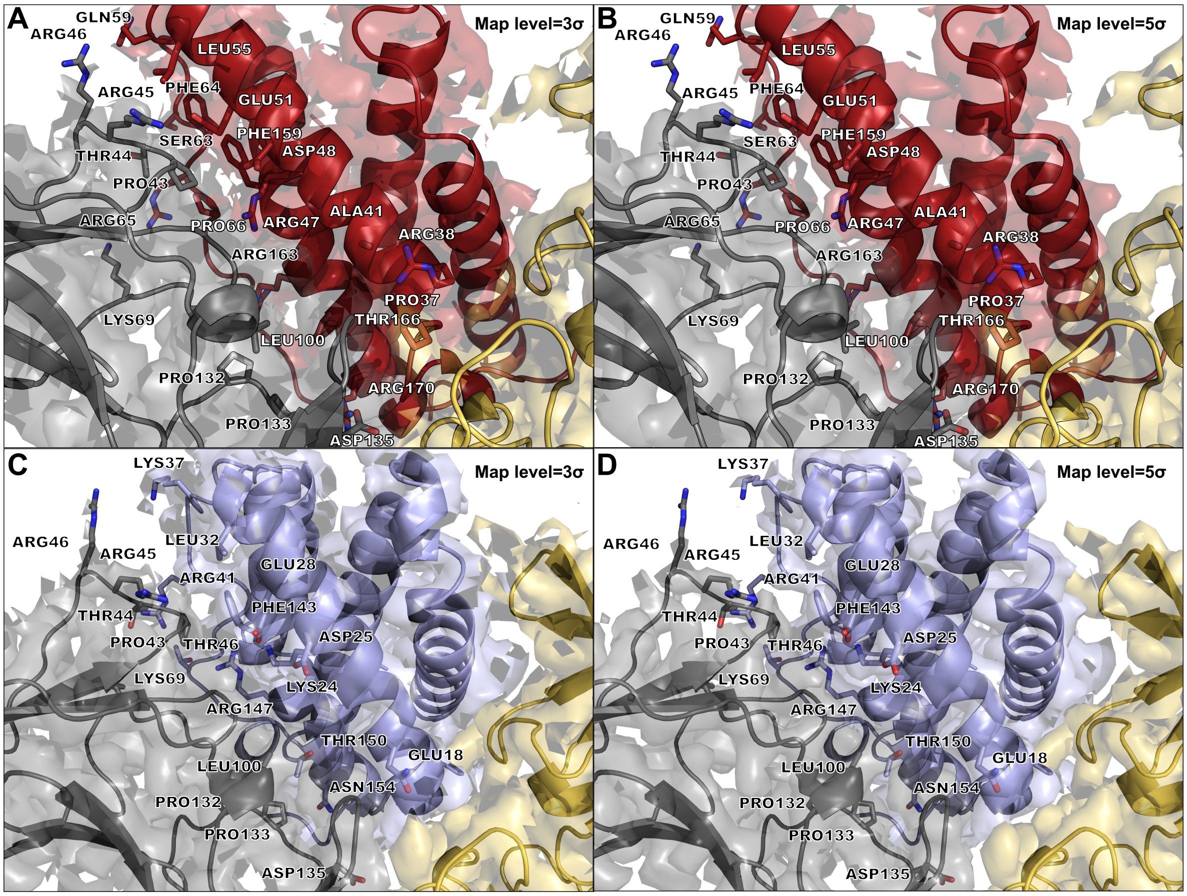


**Supplementary Figure 9: Map fitting of the residues involved in BSA calculation for IFNλ4 and IFNλ3. A) B)** Residues at IFNλR1 and IFNλ4 interface involved in BSA calculation with the cryoEM map contoured at 3σ and 5σ, respectively. **C) D)** Residues at IFNλR1 and IFNλ3 interface involved in BSA calculation with the cryoEM map contoured at 3σ and 5σ, respectively.

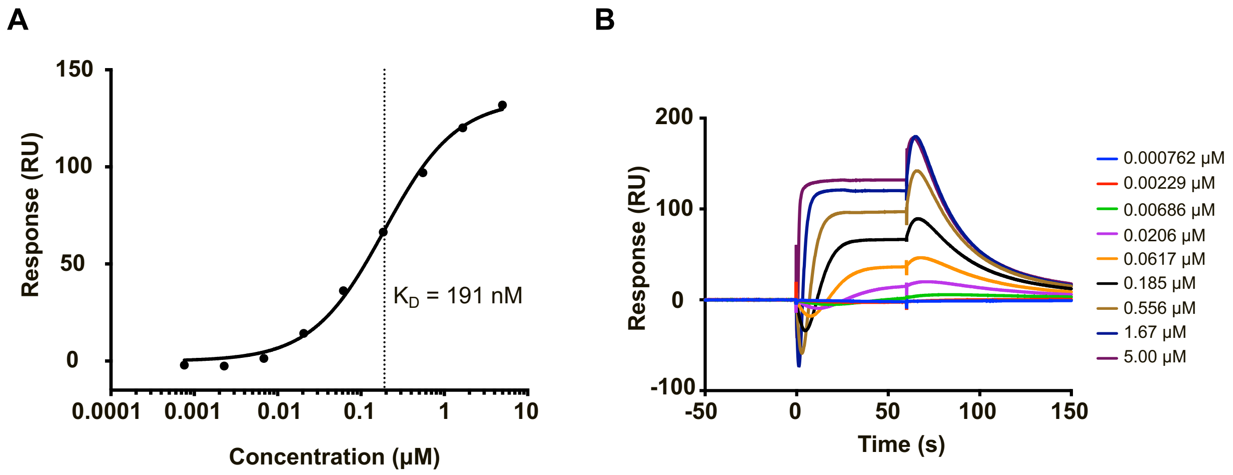


**Supplementary Figure 10: Surface plasmon resonance (SPR) to determine the affinity of the IFNλ4:IFNλR1 interaction. A)** IFNλR1 affinity for IFNλ4 ($K_D$ = 191 nM) determined by surface plasmon resonance. $K_D$ value was determined by fitting to a 1:1 binding model (Ligand on chip = IFNλR1, analyte = IFNλ4). **B)** Sensorgrams at various concentrations of IFNλ4.

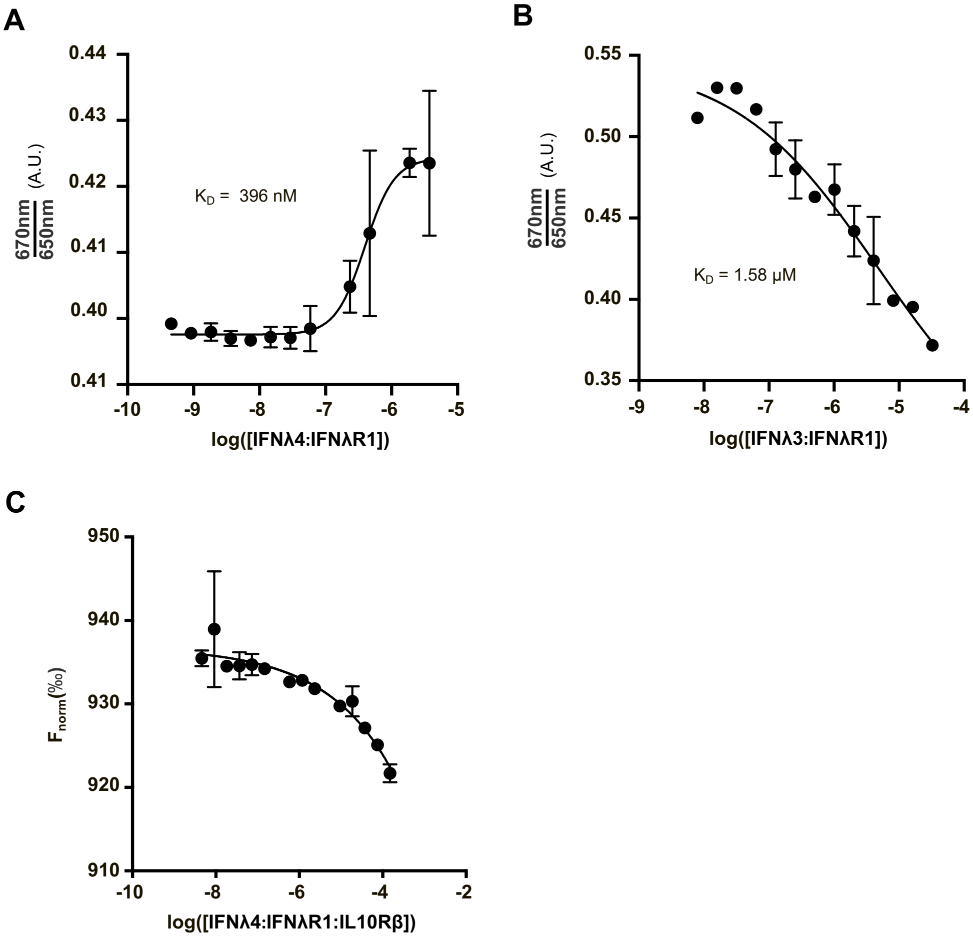


**Supplementary Figure 11: Microscale thermophoresis (MST) to calculate affinities of the IFNλ4:IFNλR1, IFNλ3:IFNλR1, and IFNλ4:IFNλR1:IL10Rβ interactions. A)** IFNλR1 affinity for IFNλ4 ($K_D$ = 396 nM) determined by microscale thermophoresis. $K_D$ value was determined by fitting to a 1:1 binding model (Confidence range = 246 nM – 1.50 μM, n = 2). **B)** IFNλR1 affinity for IFNλ3 ($K_D$ = 1.58 μM) determined by microscale thermophoresis. $K_D$ value was determined by fitting to a 1:1 binding model (Confidence range = 0.85 – 2.95 μM, n = 2). **C)** Interaction of the IFNλ4:IFNλR1:IL10Rβ complex ($K_D$ > 30 μM) (n = 2).

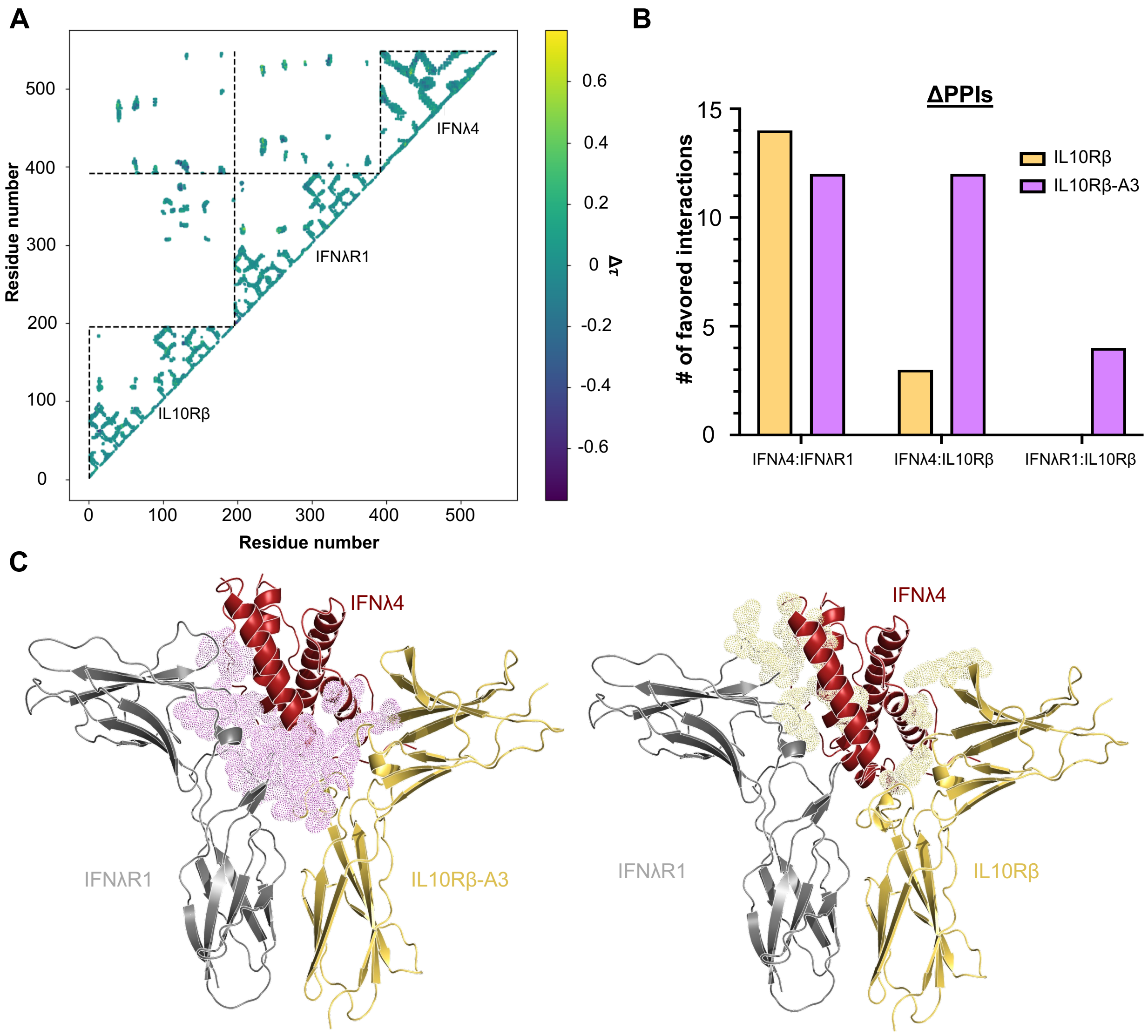


**Supplementary Figure 12: Differences in protein-protein interactions (PPIs) imparted by IL10Rβ-A3 in the IFNλ4 receptor complex. A)** Residue contact map showing residue-residue contacts that are favored for the wild-type ($\Delta\tau > 0$, yellow) or engineered ($\Delta\tau < 0$, purple) IFNλ4 complexes. **B)** Differences in protein-protein interactions (PPIs) for the engineered IFNλ4 complex relative to a model of the IFNλ4 complex with the wild-type IL10Rβ. **C)** Hotspots of interaction for the IFNλ4 receptor complex with the engineered receptor IL10Rβ-A3 (left, purple) and a model of the native IFNλ4 receptor complex (right, gold).

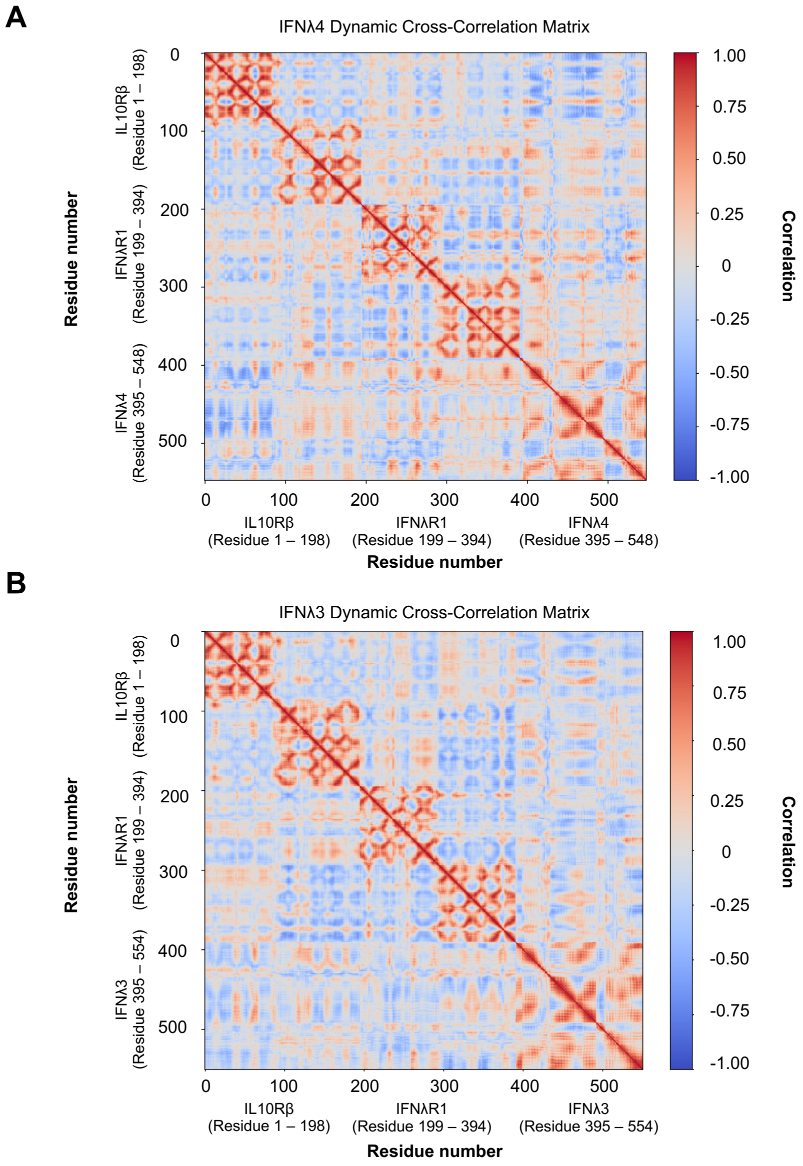


**Supplementary Figure 13: Dynamic cross-correlation (DCC) of the IFNλ4 and IFNλ3 receptor complex. A)** Dynamic cross-correlation (DCC) matrix for the IFNλ4 complex. Correlation value of 1 (red) indicates perfectly correlated residues. Correlation value of -1 (blue) indicates perfectly anti-correlated residues. **B)** Dynamic cross-correlation (DCC) matrix for the IFNλ3 complex. Correlation value of 1 (red) indicates perfectly correlated residues. Correlation value of -1 (blue) indicates perfectly anti-correlated residues.

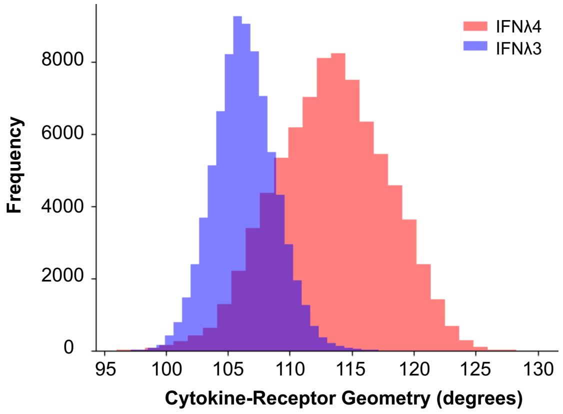


**Supplementary Figure 14: Differences in IFNλ4 and IFNλ3 receptor complex geometry persist during longer molecular dynamics (MD) simulations.** Histogram showing frequencies of cytokine-receptor geometry measurements throughout extended simulation time (3x300 ns). Angle is calculated between the center-of-masses of the D1 domain of IFNλR1, IFNλ3 (blue) or IFNλ4 (red), and the SD1 domain of IL10Rβ-A3 as shown in Figure 3.

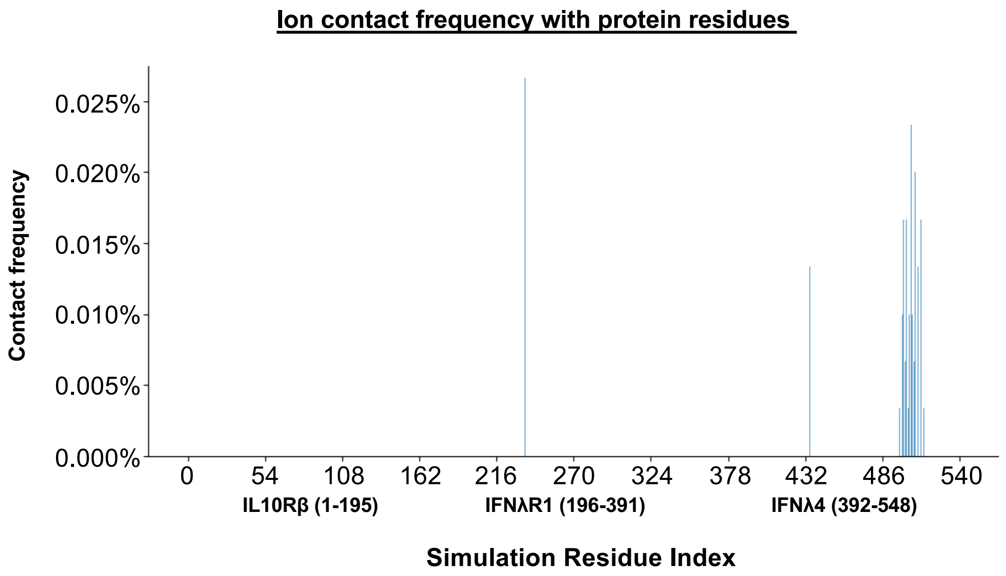


**Supplementary Figure 15: Negative ions associate only with Helix E of IFNλ4 throughout simulation time.** Ion contact frequency within 1 Å for all protein residues throughout simulation time is shown.

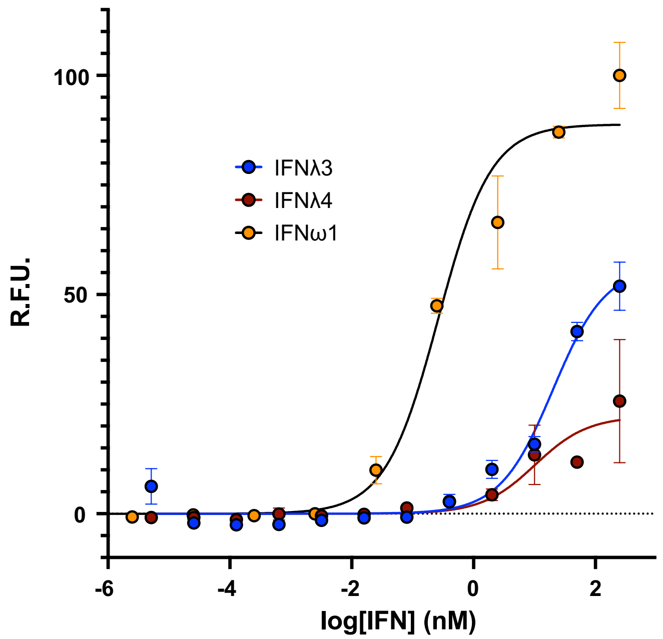


**Supplementary Figure 16: Differential activity of IFNλ4 and IFNλ3 is observed in multiple adherent cell lines.** pSTAT1 signaling of IFNω1, IFNλ3, and IFNλ4 in A549 cells. Curves are fit to a first-order logistic model. Data are presented as mean values +/- SEM (n = 3 biologically independent experiments).

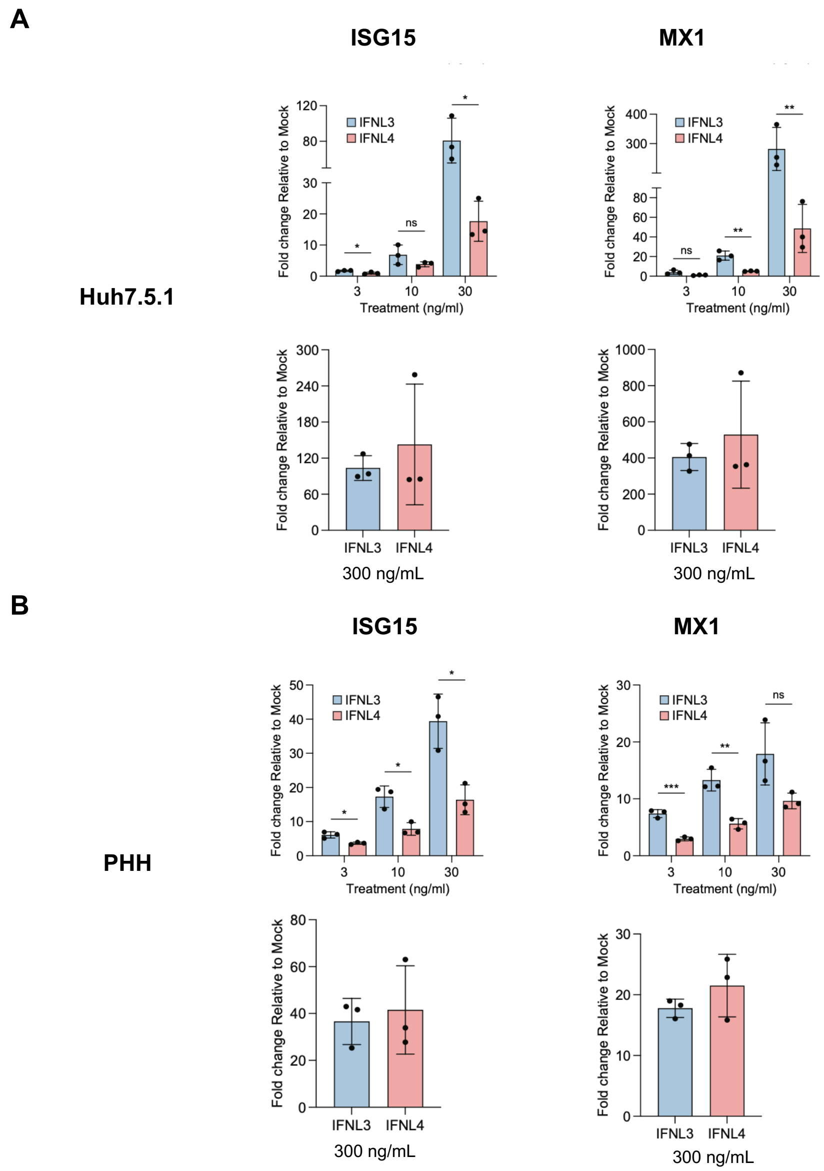


**Supplementary Figure 17: Antiviral gene induction in various cells types at multiple concentrations of IFNλ3 and IFNλ4. A)** Gene induction data for Huh7.5.1 cells. Data are presented as mean values +/- SD (n = 3 biologically independent experiments; * = $p \leq 0.05$, ** = $p \leq 0.01$). **B)** Gene induction data for primary human hepatocytes (PHH). Data are presented as mean values +/- SD (n = 3 biologically independent experiments; * = $p \leq 0.05$, ** = $p \leq 0.01$).

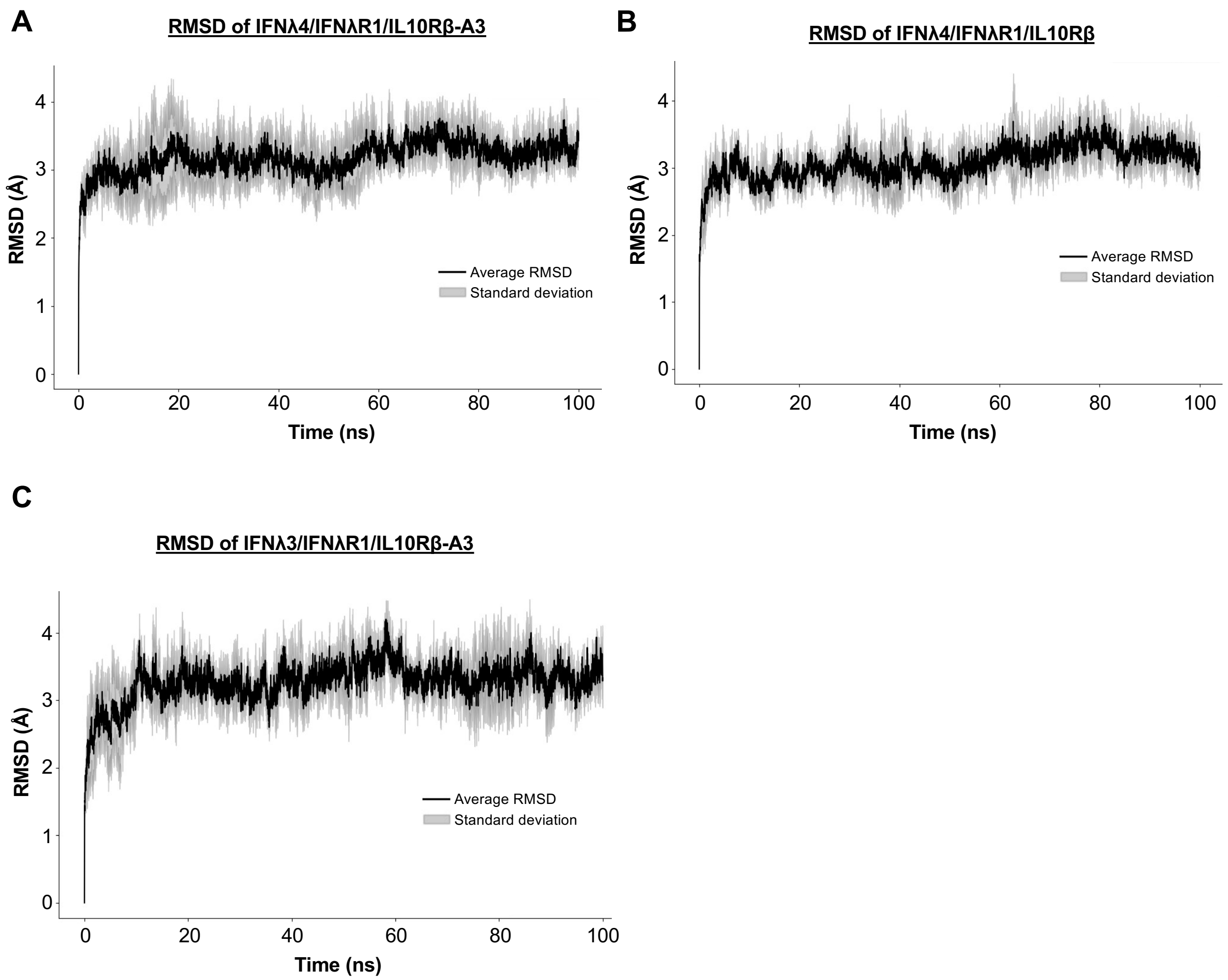


**Supplementary Figure 18: RMSD analysis of the molecular dynamics production runs. A)** RMSD analysis of the IFNλ4 receptor complex with IL10Rβ-A3. **B)** RMSD analysis of a model of the native IFNλ4 complex. **C)** RMSD analysis of the IFNλ3 receptor complex with IL10Rβ-A3.

## Supplementary Tables

| Residue | IFNλ3 residue number | IFNλ3 BSA (Å²) | IFNλ4 residue number | IFNλ4 BSA (Å²) | Fold difference |
|---|---|---|---|---|---|
| ASP | 25 | 0.13 | 48 | 12.09 | 93.00 |
| GLU | 28 | 24.52 | 51 | 40.19 | 1.64 |
| LEU | 32 | 8.3 | 55 | 39.34 | 4.74 |
| PHE | 143 | 72.35 | 159 | 35.95 | 0.50 |
| ARG | 147 | 62.59 | 163 | 40.01 | 0.64 |
| THR | 150 | 76.36 | 166 | 55.15 | 0.72 |

**Supplementary Table 1: Differences in buried surface area (BSA) for conserved residues of IFNλ4 and IFNλ3.** The fold difference is calculated by dividing IFNλ4 BSA by IFNλ3 BSA.

| IFNλ3 residue | IFNλ3 BSA (Å²) | IFNλ4 residue | IFNλ4 BSA (Å²) | Fold difference |
|---|---|---|---|---|
| - | - | PRO37 | 19.79 | 19.79 |
| GLN18 | 37.27 | ALA41 | 15.72 | 0.42 |
| LYS24 | 22.13 | ARG47 | 62.79 | 2.84 |
| - | - | GLN59 | 37.46 | 37.46 |
| LYS37 | 2.16 | - | - | 2.16 |
| ARG41 | 47.43 | - | - | 47.43 |
| - | - | SER63 | 0.84 | 0.84 |
| - | - | PHE64 | 27.54 | 27.54 |
| - | - | ARG65 | 79.36 | 79.36 |
| - | - | PRO66 | 66.95 | 66.95 |
| THR46 | 7.16 | - | - | 7.16 |
| ASN154 | 20.64 | ARG170 | 47.27 | 2.29 |

**Supplementary Table 2: Differences in buried surface area (BSA) for non-conserved residues of IFNλ4 and IFNλ3.** The fold difference is calculated by dividing IFNλ4 BSA by IFNλ3 BSA.

| Residue on IFNλR1 | IFNλ3 BSA (Å²) | IFNλ4 BSA (Å²) | Fold difference |
|---|---|---|---|
| PRO43 | 33.61 | 62.25 | 1.85 |
| THR44 | 12.04 | 54.44 | 4.52 |
| ARG45 | 8.64 | 61.14 | 7.08 |
| ARG46 | 2.32 | 42.38 | 18.27 |
| LYS69 | 7.52 | 17.33 | 2.30 |
| LEU100 | 14.71 | 6.53 | 0.44 |
| PRO132 | 0.93 | 2.34 | 2.52 |
| PRO133 | 61.68 | 79.19 | 1.28 |
| ASP135 | 11.5 | 23.13 | 2.01 |

**Supplementary Table 3: Differences in buried surface area (BSA) for IFNλR1 residues engaging with IFNλ4 and IFNλ3.** The fold difference is calculated by dividing IFNλ4 BSA by IFNλ3 BSA.

| Protein 1 | Protein 2 | Residue 1 | Residue 2 | $\Delta\tau$ |
|---|---|---|---|---|
| IFNλ4 | IL10Rβ-A3 | GLU36 | THR144 | 0.7677 |
| IFNλ4 | IL10Rβ-A3 | PRO37 | THR142 | 0.6951 |
| IFNλ4 | IL10Rβ-A3 | LEU35 | TRP143 | 0.689 |
| IFNλ4 | IFNλR1 | ARG67 | ASP71 | 0.6874 |
| IFNλ4 | IFNλR1 | ARG170 | PRO133 | 0.6483 |
| IFNλ4 | IFNλR1 | PRO66 | LEU72 | 0.6369 |
| IFNλ4 | IL10Rβ-A3 | ARG38 | TYR140 | 0.6225 |
| IFNλ4 | IFNλR1 | LEU171 | PRO133 | 0.5998 |
| IFNλ4 | IL10Rβ-A3 | SER34 | TRP143 | 0.5724 |
| IFNλ4 | IL10Rβ-A3 | ARG38 | ASN138 | 0.5423 |
| IFNλ4 | IFNλR1 | ARG67 | TYR73 | 0.5212 |
| IFNλR1 | IL10Rβ-A3 | ASP135 | ASP147 | 0.5099 |
| IFNλ4 | IFNλR1 | ARG163 | PRO132 | 0.5012 |
| IFNλR1 | IL10Rβ-A3 | VAL186 | THR142 | 0.4941 |
| IFNλ4 | IFNλR1 | THR166 | PRO133 | 0.4925 |
| IFNλ4 | IFNλR1 | SER34 | LEU134 | 0.4871 |
| IFNλ4 | IFNλR1 | THR166 | PRO132 | 0.4838 |
| IFNλ4 | IFNλR1 | ARG33 | LEU134 | 0.4781 |
| IFNλR1 | IL10Rβ-A3 | SER185 | TYR140 | 0.464 |
| IFNλ4 | IFNλR1 | ARG170 | LEU134 | 0.4584 |
| IFNλ4 | IL10Rβ-A3 | GLU36 | TYR140 | 0.4574 |
| IFNλ4 | IFNλR1 | ARG65 | GLN70 | 0.4457 |
| IFNλR1 | IL10Rβ-A3 | PHE184 | TRP143 | 0.4437 |
| IFNλ4 | IL10Rβ-A3 | ALA113 | ASP84 | 0.4363 |
| IFNλ4 | IL10Rβ-A3 | TYR32 | ASP147 | 0.4339 |
| IFNλ4 | IL10Rβ-A3 | PRO37 | TRP143 | 0.432 |
| IFNλ4 | IL10Rβ-A3 | PRO107 | GLY83 | 0.4223 |
| IFNλ4 | IL10Rβ-A3 | PRO107 | TYR82 | 0.4132 |

**Supplementary Table 4: Protein-protein interactions favored by the IFNλ4/IFNλR1/IL10Rβ-A3 complex.** A table detailing protein-protein interactions, specific residue-residue interactions, and the magnitudes of differences in residue contact time ($\Delta\tau$) are provided. Data represent protein-protein interactions ranking in the top 50 of overall contact differences.

| Protein 1 | Protein 2 | Residue 1 | Residue 2 | $\Delta\tau$ |
|---|---|---|---|---|
| IFNλ4 | IFNλR1 | SER156 | THR44 | 0.7686 |
| IFNλ4 | IFNλR1 | ALA155 | THR44 | 0.6208 |
| IFNλ4 | IFNλR1 | PHE64 | ARG45 | 0.6185 |
| IFNλ4 | IFNλR1 | SER63 | ARG46 | 0.6015 |
| IFNλ4 | IFNλR1 | CYS152 | THR44 | 0.5992 |
| IFNλ4 | IL10Rβ | HIS31 | LYS81 | 0.5864 |
| IFNλ4 | IFNλR1 | GLY58 | ARG46 | 0.5851 |
| IFNλ4 | IFNλR1 | ARG65 | ARG45 | 0.5764 |
| IFNλ4 | IFNλR1 | ALA155 | PRO43 | 0.5693 |
| IFNλ4 | IFNλR1 | LEU161 | ASN74 | 0.548 |
| IFNλ4 | IFNλR1 | SER63 | ARG45 | 0.5166 |
| IFNλ4 | IFNλR1 | ASN160 | PRO43 | 0.4972 |
| IFNλ4 | IFNλR1 | LEU164 | TYR73 | 0.461 |
| IFNλ4 | IFNλR1 | GLY58 | ARG45 | 0.459 |
| IFNλ4 | IL10Rβ | ALA113 | TYR59 | 0.445 |
| IFNλ4 | IFNλR1 | SER156 | PRO43 | 0.436 |
| IFNλ4 | IL10Rβ | GLU110 | ARG60 | 0.4266 |

**Supplementary Table 5: Protein-protein interactions favored by a model of the IFNλ4/IFNλR1/IL10Rβ complex.** A table detailing protein-protein interactions, specific residue-residue interactions, and the magnitudes of differences in residue contact time ($\Delta\tau$) are provided. Data represent protein-protein interactions ranking in the top 50 of overall contact differences.

| Protein | Genetic sequence |
|---|---|
| IFNλ4 (Sequence1) | GCTCCAAGAAGATGTTTGTTGTCTCACTACAGATCTTTGGAACCAAGAA<br>CTTTGGCTGCTGCTAAGGCTTTGAGAGACAGATACGAAGAAGAAGCTTT<br>GTCTTGGGGTCAAAGAAACTGTTCTTTCAGACCAAGAAGAGACCCACCA<br>AGACCATCTTCTTGTGCTAGATTGAGACACGTTGCTAGAGGTATCGCTGA<br>CGCTCAAGCTGTTTTGTCTGGTTTGCACAGATCTGAATTGTTGCCAGGTG<br>CTGGTCCAATCTTGGAATTGTTGGCTGCTGCTGGTAGAGACGTTGCTGCT<br>TGTTTGGAATTGGCTAGACCAGGTTCTTCTAGAAAGGTTCCAGGTGCTCA<br>AAAGAGAAGACACAAGCCAAGAAGAGCTGACTCTCCAAGATGTAGAAA<br>GGCTTCTGTTGTTTTCAACTTGTTGAGATTGTTGACTTGGGAATTGAGAT<br>TGGCTGCTCACTCTGGTCCATGTTTG |
| IL10Rβ-IFNλ4 (Sequence2) | ATGGTACCACCTCCCGAAAATGTCAGAATGAATTCTGTTAATTTCAAGA<br>ACATTCTACAGTGGGAGTCACCTGCTTTTGCCAAAGGGAACCTGACTTTC<br>ACAGCTCAGTACCTAAGTTATAGGATATTCCAAGATAAATGCATGAATA<br>CTACCTTGACGGAATGTGATTTCTCAAGTCTTTCCAAGTATGGTGACCAC<br>ACCTTGAGAGTCAGGGCTGAATTTGCAGATGAGCATTCAGACTGGGTAA<br>ACATCACCTTCTGTCCTGTGGATGACACCATTATTGGACCCCCTGGAATG<br>CAAGTAGAAGTACTTGCTGATTCTTTACATATGCGTTTCTTAGCCCCTAA<br>AATTGAGAATGAATACGAAACTTGGACTATGAAGAATGTGTATAACTCA<br>TGGACTTATAATGTGCAATACTGGAAAAACGGTACTGATGAAAAGTTTC<br>AAATTACTCCCCAGTATGACTTTGAGGTCCTCAGAAACCTGGAGCCATG<br>GACAACTTATTGTGTTCAAGTTCGAGGGTTTCTTCCTGATCGGAACAAAG<br>CTGGGGAATGGAGTGAGCCTGTCTGTGAGCAAACAACCCATGACGAAAC<br>GGTCCCCTCCGGTGGTGGTGGATCTTTGGAAGTTCTGTTTCAAGGGCCTG<br>GAGGTGGTTCTGAAGGTGGAGGATCTGGAGCGGCC+Sequence1 |
| sfGFP-IL10Rβ-IFNλ4 (Sequence3) | ATGAGCAAAGGTGAAGAACTGTTTACCGGCGTTGTGCCGATTCTGGTGG<br>AACTGGATGGCGATGTGAACGGTCACAAATTCAGCGTGCGTGGTGAAGG<br>TGAAGGCGATGCCACGATTGGCAAACTGACGCTGAAATTTATCTGCACC<br>ACCGGCAAACTGCCGGTGCCGTGGCCGACGCTGGTGACCACCCTGACCT<br>ATGGCGTTCAGTGTTTTAGTCGCTATCCGGATCACATGAAACGTCACGAT<br>TTCTTTAAATCTGCAATGCCGGAAGGCTATGTGCAGGAACGTACGATTA<br>GCTTTAAAGATGATGGCAAATATAAAACGCGCGCCGTTGTGAAATTTGA<br>AGGCGATACCCTGGTGAACCGCATTGAACTGAAAGGCACGGATTTTAAA<br>GAAGATGGCAATATCCTGGGCCATAAACTGGAATACAACTTTAATAGCC<br>ATAATGTTTATATTACGGCGGATAAACAGAAAAATGGCATCAAAGCGAA<br>TTTTACCGTTCGCCATAACGTTGAAGATGGCAGTGTGCAGCTGGCAGAT<br>CATTATCAGCAGAATACCCCGATTGGTGATGGTCCGGTGCTGCTGCCGG<br>ATAATCATTATCTGAGCACGCAGACCGTTCTGTCTAAAGATCCGAACGA<br>AAAAGGCACGCGGGACCACATGGTTCTGCACGAATATGTGAATGCGGCA<br>GGTATTACGGGATCA+Sequence2 |

**Supplementary Table 6: Genetic sequences of IFNλ4 constructs**

| Primer | Sequence |
|---|---|
| ISG15_fwd | CGCAGATCACCCAGAAGATCG |
| ISG15_rev | TTCGTCGCATTTGTCCACCA |
| MX1_fwd | GTTTCCGAAGTGGACATCGCA |
| MX1_rev | CTGCACAGGTTGTTCTCAGC |
| APOL3_fwd | GGGACGAGTCTGGCCCTTA |
| APOL3_rev | TCAATCGGTCAATGCTGGTTG |
| SAMD9L_fwd | ATTCCAAGCAACGGGATGTAG |
| SAMD9L_rev | AGTCTCGGTTTCCTATGAGAAGT |
| 18S_fwd | GTAACCCGTTGAACCCCATT |
| 18S_rev | CCATCCAATCGGTAGTAGCG |

**Supplementary Table 7: DNA primers used for quantification of gene induction in Hap1 cells.**

| Primer | Sequence |
|---|---|
| ISG15_fwd | AGATCACCCAGAAGATCGGC |
| ISG15_rev | TTCGTCGCATTTGTCCACCA |
| MX1_fwd | CTGGTGCTGAAACTGAAGAAAC |
| MX1_rev | TACCTCTGAAGCATCCGAAATC |
| GAPDH_fwd | GGCTGAGAACGGGAAGCTTGTCAT |
| GAPDH_rev | CAGCCTTCTCCATGGTGGTGAAGA |
| PCR_fwd | CTAGCCATGGCGTTAGTATGAG |
| PCR_rev | CGGGCATAGAGTGGGTTTATC |
| Probe | FAM-5’-CCGGGAAGACTGGGTCCTTTCTTG-3’-TAMRA |

**Supplementary Table 8: DNA primers used for quantification of gene induction in Huh7.5.1 cells and PHHs.**

| | IFNλ4 | IFNλ3 |
|---|---|---|
| **Data collection and processing** | | |
| Microscope | Krios (University of Chicago) | Krios (University of Chicago) |
| Magnification | 81,000 | 81,000 |
| Voltage (kV) | 300 | 300 |
| Spherical aberration (mm) | 2.7 | 2.7 |
| Detector | K3 | K3 |
| Camera mode | Super resolution counting | Super resolution counting |
| Exposure rate ($e^-$/pixel/s) | 15 | 15 |
| Total exposure ($e^-$/Å$^2$) | 60 | 60 |
| Defocus range (μm) | -0.9 to -2.3 | -0.9 to -2.3 |
| Pixel size (Å) | 1.068 | 1.065 |
| Mode of data collection | Image shift | Image shift |
| Energy filter | 20 eV slit | 20 eV slit |
| Software for data collection | EPU | EPU |
| Number of micrographs | 5,130 | 2,928 |
| **Refinement** | | |
| **Model resolution (Å)** | 3.26 | 3.00 |
| FSC threshold | 0.143 | 0.143 |
| **Map sharpening B factor (Å$^2$)** | -95.43 | -85.22 |
| **Model composition** | | |
| Non-hydrogen atoms | 4319 | 4417 |
| Protein residues | 529 | 542 |
| Ligands | 5 | 2 |
| **B factors (Å$^2$)** | | |
| Protein | 57.31 | 59.42 |
| Ligand | 43.83 | 74.00 |
| **R.m.s. deviations** | | |
| Bond lengths (Å) | 0.005 | 0.008 |
| Bond angles (°) | 0.745 | 1.120 |
| **Validation** | | |
| MolProbity score | 1.82 | 1.46 |
| Clashscore | 8.90 | 8.58 |
| Poor rotamers (%) | 0.64 | 0.61 |
| **Ramachandran plot** | | |
| Favored (%) | 95.01 | 98.13 |
| Allowed (%) | 4.99 | 1.87 |
| Disallowed (%) | 0 | 0 |

**Supplementary Table 9: Information regarding data collection, processing, and refinement for cryoEM of the IFNλ4 and IFNλ3 ternary complexes.**

| System Components | IFNλ4/IFNλR1/IL10Rβ-A3 | IFNλ4/IFNλR1/IL10Rβ | IFNλ3/IFNλR1/IL10Rβ |
|---|---|---|---|
| Protein Chains | 3 chains (A, B, C) | 3 chains (A, B, C) | 3 chains (A, B, C) |
| Water Molecules | 39,045 | 39,046 | 33,483 |
| Ions | 10 $Cl^-$ | 10 $Cl^-$ | 5 $Na^+$ |
| Total Number of Atoms | 125,898 | 125,902 | 109,285 |
| Box type | dodecahedron | dodecahedron | dodecahedron |
| Box size | 12.18x12.18x8.62 $nm^3$ | 12.18x12.18x8.62 $nm^3$ | 11.62x11.62x8.22 $nm^3$ |

**Supplementary Table 10: System components and dimensions for molecular dynamics simulations**

| Phase | Duration | Parameters |
| --- | --- | --- |
| **NVT Equilibration** | 500 ps | T = 300K (V-rescale)<br>2 fs timestep<br>Position restraints on protein |
| **NPT Equilibration** | 5 ns | T = 300K (V-rescale)<br>2 fs timestep<br>P = 1 bar (Berendsen)<br>Position restraints on protein |
| **Production MD** | 100 ns (300 ns for runs in SI) | T = 300K (Nose-Hoover)<br>2 fs timestep<br>P = 1 bar (Parrinello-Rahman)<br>No position restraints |

**Supplementary Table 11: Simulation protocols for molecular dynamics simulations**

| Parameter | Value |
|---|---|
| **Constraints** | |
| - Algorithm | LINCS |
| - Type | H-bonds |
| - LINCS order/iterations | 4/1 |
| **Non-bonded Interactions** | |
| - Coulomb cutoff | 1.2 nm |
| - VdW cutoff | 1.2 nm |
| - PME order | 4 |
| - Fourier spacing | 0.16 nm |
| **Output Frequency (Production)** | |
| - Coordinates | Every 10 ps |
| - Energies | Every 10 ps |
| - Log | Every 10 ps |

**Supplementary Table 12: Technical parameters for molecular dynamics simulations**
