# Supplementary material for: Structural studies of the IFNλ4 receptor complex using cryoEM enabled by protein engineering
Source: Nat Commun. 2025 Jan 18;16:818. doi: 10.1038/s41467-025-56119-y (PMC11742915; doi:10.1038/s41467-025-56119-y)
Supplement: Supplementary file 2 — Reporting Summary [file 41467_2025_56119_MOESM2_ESM.pdf]

nature portfolio

Corresponding author(s): Mendoza

Last updated by author(s): Dec 8, 2024

Reporting Summary

Nature Portfolio wishes to improve the reproducibility of the work that we publish. This form provides structure for consistency and transparency in reporting. For further information on Nature Portfolio policies, see our Editorial Policies and the Editorial Policy Checklist.

Statistics

For all statistical analyses, confirm that the following items are present in the figure legend, table legend, main text, or Methods section.

n/a	Confirmed	
☐	☒	The exact sample size (*n*) for each experimental group/condition, given as a discrete number and unit of measurement
☐	☒	A statement on whether measurements were taken from distinct samples or whether the same sample was measured repeatedly
☐	☒	The statistical test(s) used AND whether they are one- or two-sided<br>*Only common tests should be described solely by name; describe more complex techniques in the Methods section.*
☒	☐	A description of all covariates tested
☒	☐	A description of any assumptions or corrections, such as tests of normality and adjustment for multiple comparisons
☐	☒	A full description of the statistical parameters including central tendency (e.g. means) or other basic estimates (e.g. regression coefficient) AND variation (e.g. standard deviation) or associated estimates of uncertainty (e.g. confidence intervals)
☐	☒	For null hypothesis testing, the test statistic (e.g. *F*, *t*, *r*) with confidence intervals, effect sizes, degrees of freedom and *P* value noted<br>*Give P values as exact values whenever suitable.*
☒	☐	For Bayesian analysis, information on the choice of priors and Markov chain Monte Carlo settings
☒	☐	For hierarchical and complex designs, identification of the appropriate level for tests and full reporting of outcomes
☒	☐	Estimates of effect sizes (e.g. Cohen's *d*, Pearson's *r*), indicating how they were calculated

Our web collection on statistics for biologists contains articles on many of the points above.

Software and code

Policy information about availability of computer code

Data collection	For cell signaling, flow cytometry data was collected using a Beckman-Coulter CytoFlex S flow cytometer running CytExpert version 2.5. Molecular simulation data was generated using GROMACS 2020.5 with the AMBER ff99sb-ILDN* force field. Structural data was collected using a Vitrobot Mark IV (Thermo).
Data analysis	All statistical analyses were performed using Prism (GraphPad version 9). Molecular simulation data was analyzed using Python. Structural data was processed and analyzed using CryoSPARC live, RELION 5.0 beta, PHENIX, and Coot - extensive additional details on structure data analysis are found in the Methods section of the manuscript. For further information regarding scripts used for analysis, please visit https://github.com/bylehn/ifnl4-structure-paper for all additional computational resources relevant to this project.

For manuscripts utilizing custom algorithms or software that are central to the research but not yet described in published literature, software must be made available to editors and reviewers. We strongly encourage code deposition in a community repository (e.g. GitHub). See the Nature Portfolio guidelines for submitting code & software for further information.

Data

Policy information about availability of data

All manuscripts must include a data availability statement. This statement should provide the following information, where applicable:

- Accession codes, unique identifiers, or web links for publicly available datasets
- A description of any restrictions on data availability
- For clinical datasets or third party data, please ensure that the statement adheres to our policy

Structure factors and coordinates have been deposited to the Protein Data Bank (9BPU, 9BPV) and the EMBO (EMD-44790, EMD-44791). All relevant computational resources for this project can be found at https://github.com/bylehn/ifnl4-structure-paper . All data relevant to the conclusions presented within the manuscript are included, with additional data available upon further request.

Research involving human participants, their data, or biological material

Policy information about studies with human participants or human data. See also policy information about sex, gender (identity/presentation), and sexual orientation and race, ethnicity and racism.

Reporting on sex and gender	N/A
Reporting on race, ethnicity, or other socially relevant groupings	N/A
Population characteristics	N/A
Recruitment	N/A
Ethics oversight	N/A

Note that full information on the approval of the study protocol must also be provided in the manuscript.

Field-specific reporting

Please select the one below that is the best fit for your research. If you are not sure, read the appropriate sections before making your selection.

[x] Life sciences [ ] Behavioural & social sciences [ ] Ecological, evolutionary & environmental sciences

For a reference copy of the document with all sections, see nature.com/documents/nr-reporting-summary-flat.pdf

Life sciences study design

All studies must disclose on these points even when the disclosure is negative.

Sample size	Biological replicates for Figure 5 represent three biologically independent experiments to produce sufficient statistics for analysis.
Data exclusions	No data was excluded from this study.
Replication	All experimental results presented were analyzed in triplicate and were successful in reproducing results.
Randomization	Randomization is not relevant to the experimental outcomes of this manuscript.
Blinding	Blinding is not relevant to the experimental outcomes of this manuscript.

Reporting for specific materials, systems and methods

We require information from authors about some types of materials, experimental systems and methods used in many studies. Here, indicate whether each material, system or method listed is relevant to your study. If you are not sure if a list item applies to your research, read the appropriate section before selecting a response.

Materials & experimental systems

n/a	Involved in the study
☐	☒ Antibodies
☐	☒ Eukaryotic cell lines
☒	☐ Palaeontology and archaeology
☒	☐ Animals and other organisms
☒	☐ Clinical data
☒	☐ Dual use research of concern
☒	☐ Plants

Methods

n/a	Involved in the study
☒	☐ ChIP-seq
☐	☒ Flow cytometry
☒	☐ MRI-based neuroimaging

Antibodies

Antibodies used: Anti-Myc antibody conjugated to Alexa647 used for yeast experiments was obtained from Cell Signaling (#2233) (1:50 dilution prepared according to manufacturer specifications). Anti-Y701 pSTAT1 monoclonal antibody was obtained from Cell Signaling (#9174S) (1:50 dilution according to manufacturer specifications).

Validation: All antibodies were validated by the manufacturer prior to use (https://www.cellsignal.com/).

Eukaryotic cell lines

Policy information about cell lines and Sex and Gender in Research

Cell line source(s): HEK293 GnTi- (ATCC), HAP1 cells were a gift from Jan Carette at Stanford University, A549 cells were a gift from Curt Horvath at Northwestern University, and Huh7.5.1 cells were provided by F.V. Chisari at The Scripps Research Institute.

Authentication: All cells were authenticated by the manufacturer prior to use.

Mycoplasma contamination: Cell lines were not tested for mycoplasma contamination.

Commonly misidentified lines (See ICLAC register): There are no commonly misidentified lines used in this study.

Plants

Seed stocks: N/A

Novel plant genotypes: N/A

Authentication: N/A

Flow Cytometry

Plots

Confirm that:

☒ The axis labels state the marker and fluorochrome used (e.g. CD4-FITC).

☐ The axis scales are clearly visible. Include numbers along axes only for bottom left plot of group (a 'group' is an analysis of identical markers).

☐ All plots are contour plots with outliers or pseudocolor plots.

☐ A numerical value for number of cells or percentage (with statistics) is provided.

Methodology

Sample preparation: Hap1 cells were plated in a 96-well plate and treated with serial dilutions of either IFNλ4, IFNλ3, or IFNω1 for 15 minutes at 37o C. After removing the supernatant, cells were then treated with trypsin (Gibco) for 5 minutes and resuspended gently. Following resuspension, cells were added to 16% PFA (Electron Microscopy Services) to fix for 10 minutes at room temperature before adding ice-cold methanol to permeabilize cells. Samples were stored at -80o C until use. To quantify activation of pSTAT1, cells were washed thrice with 0.5% PBSA and then stained with an anti-Y701 pSTAT1 monoclonal

	antibody (Cell Signaling, product #9174S) according to manufacturer instructions. After incubation at 4o C for at least 15 minutes with gentle rotation, samples were analyzed using a CytoFlex S flow cytometer (Beckman Coulter). Data were analyzed in Prism (GraphPad version 9).
Instrument	Beckman-Coulter Cytoflex X
Software	CytExpert 2.5, Prism version 9
Cell population abundance	Cell lines were used for signaling experiments so there was not gating for specific populations other than removing debris and doublets.
Gating strategy	Dead cells and debris were gated out by Forward and Side scatter

☐ Tick this box to confirm that a figure exemplifying the gating strategy is provided in the Supplementary Information.
