# Supplementary material for: Structural studies of the IFNλ4 receptor complex using cryoEM enabled by protein engineering
Source: Nat Commun. 2025 Jan 18;16:818. doi: 10.1038/s41467-025-56119-y (PMC11742915; doi:10.1038/s41467-025-56119-y)

**Interface 1 – IFNL3:IL10RB_A3 (C:A)**


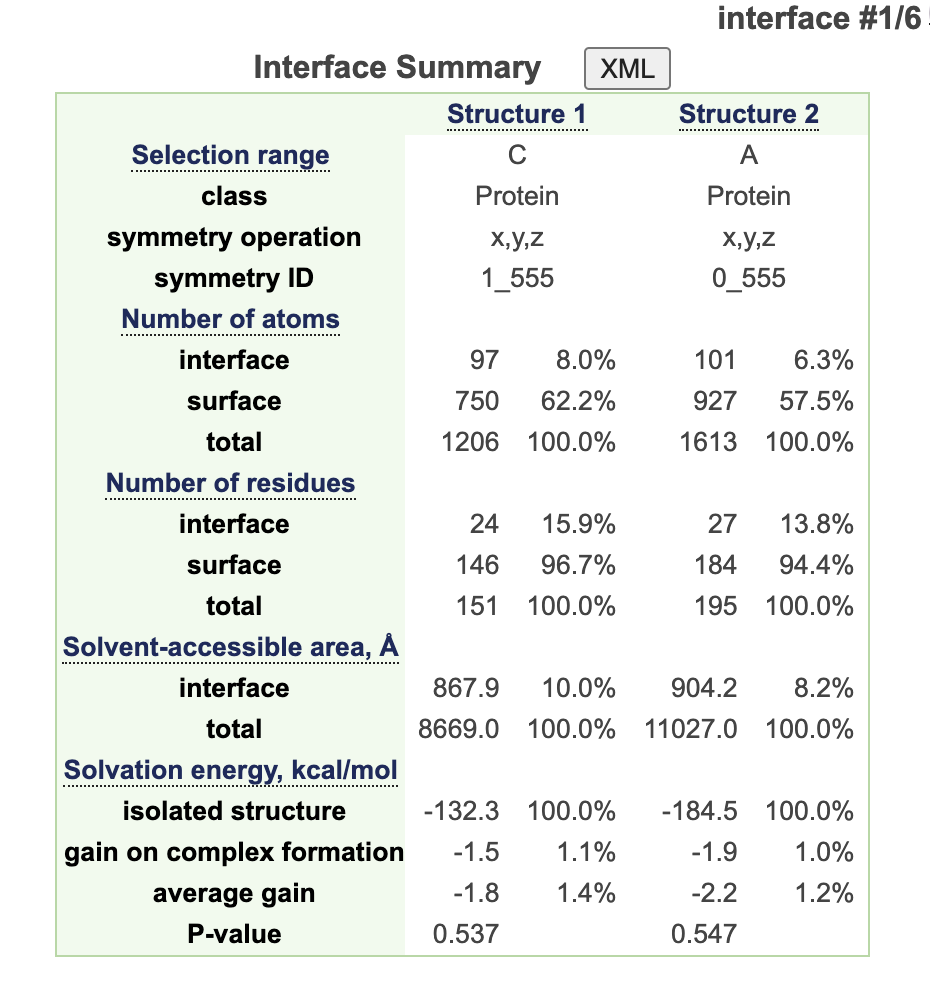


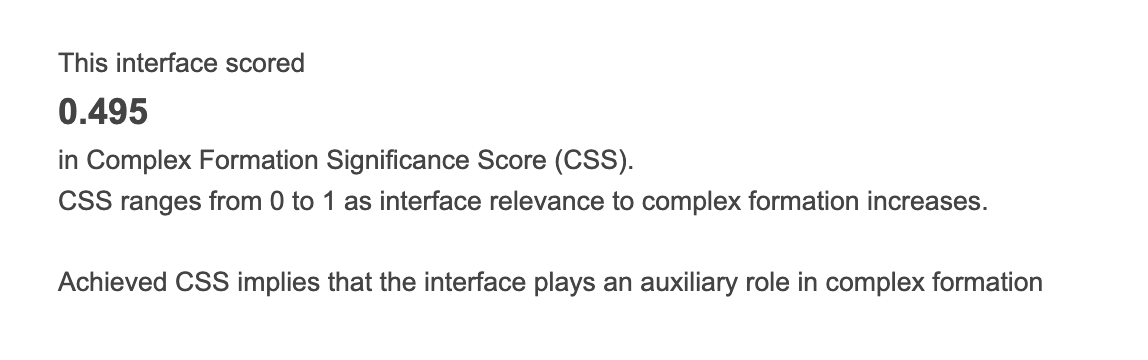


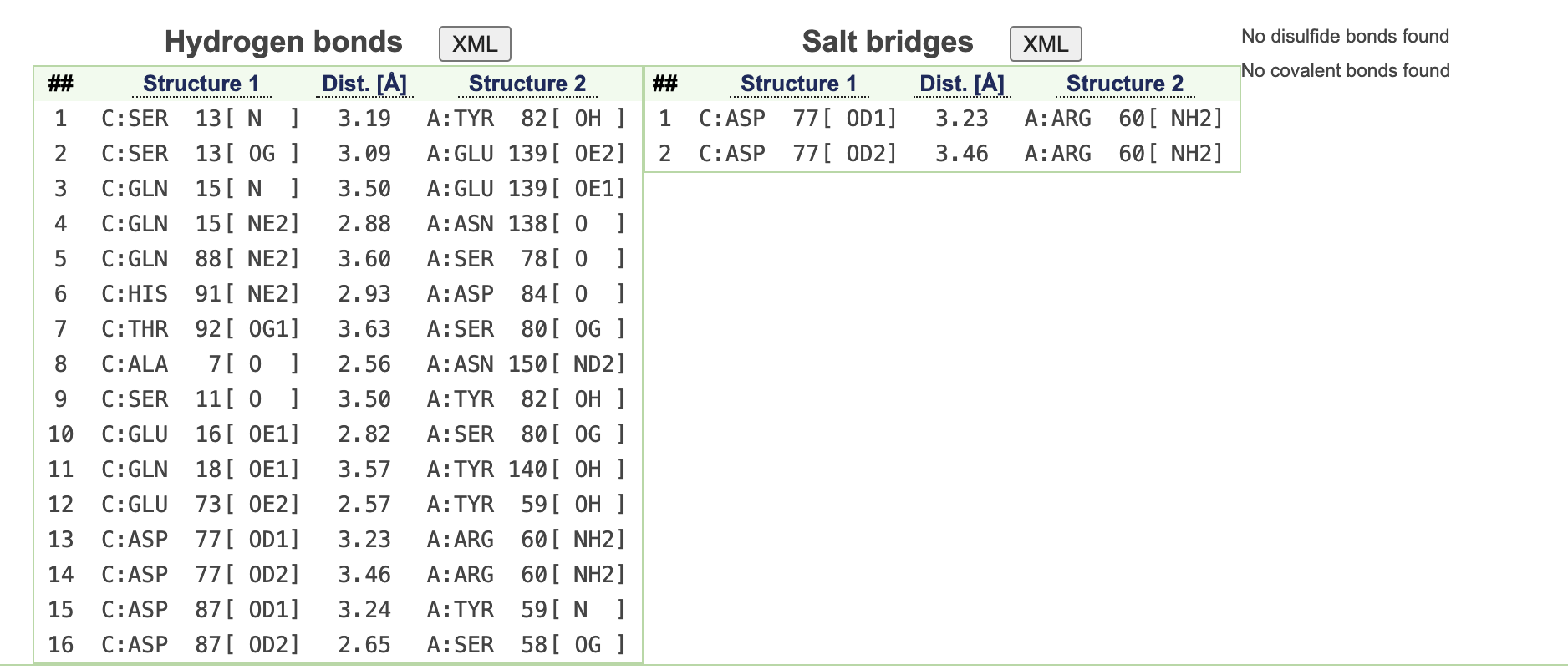


Notes:

IFNL3: 24/151 (15.9%), IL10RB_A3: 27/195 (13.8%)

Interface scored 0.495, implying that the interface plays an auxiliary role in complex formation

IFNL3: 867.9 A interface, 8669 A total (10%)

IL10RB_A3: 904.2 A interface, 11027 A total (8.2%)

Total surface area = 1772.1

18 reported interactions – 16 H-bonds and 2 salt bridges. 16 total interactions. Reported as IFNL3:IL10RB_A3.

Ala7:Asn150 (H-bond, O:ND2)

Ser11:Tyr82 (H-bond, O:OH)

Ser13:Tyr82 (H-bond, N:OH)

Ser13:Glu139 (H-bond, OG:OE2)

Gln15:Asn138 (H-bond, NE2:O)

Gln15:Asn139 (H-bond, N:OE1)

Glu16:Ser80 (H-bond, OE1:OG)

Gln18:Tyr140 (H-bond, OE1:OH)

Glu73:Tyr59 (H-bond, OE2:OH)

Asp77:Arg60 (H-bond, OD1:NH2) / Asp77:Arg60 (salt bridge, OD1:NH2)

Asp77:Arg60 (H-bond, OD2:NH2) / Asp77:Arg60 (salt bridge, OD2:NH2)

Asp87:Ser58 (H-bond, OD2:OG)

Asp87:Tyr59 (H-bond, OD1:N)

Gln88:Ser78 (H-bond, NE2:O)

His91:Asp84 (H-bond, NE2:O)

Thr92:Ser80 (H-bond, OG1:OG)

Stretches of interaction –

IFNL3: His5-Gln18 (6, 17 not included), Leu69, Glu73, Ala76-Asp77, Asp84, Asp87-His95 (89, 93 not included)

IL10RB_A3: Tyr56-Gln63 (62 not included), Ser78-His85, Val108, Asn138-Asn150 (141, 144-146 not included), Asp197-Arg198

Engineered residues (for comparison to H11 and WT IL10RB) –

Gln15 and Glu73 on IFNL3 form H-bonds with Asn138/Asn139 and Tyr59, respectively and are both ~50% buried at the interface. Asp147 (<10% buried) and Met148 (>80% buried) on A3 are at the interface, but do not bond.

All data –


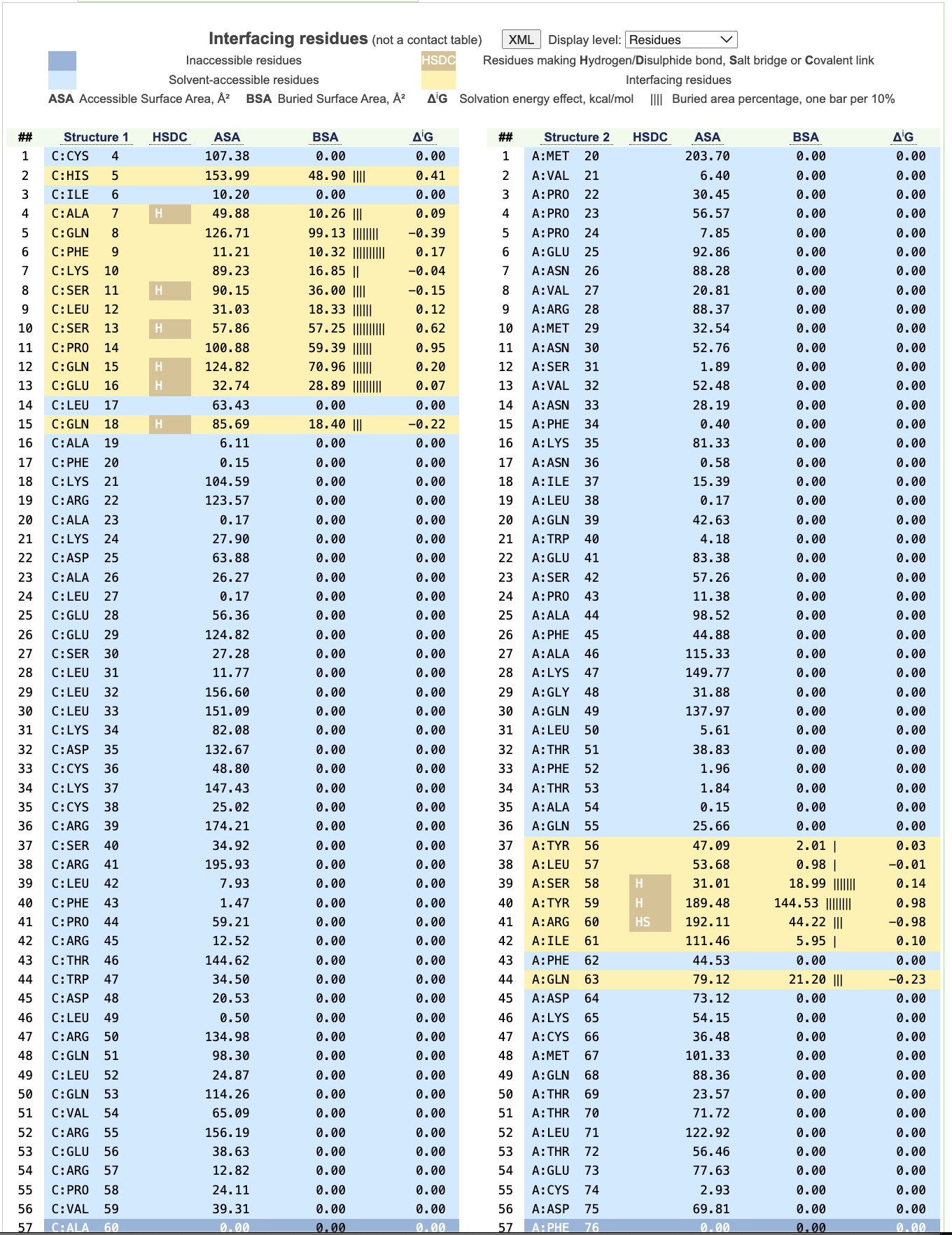


**
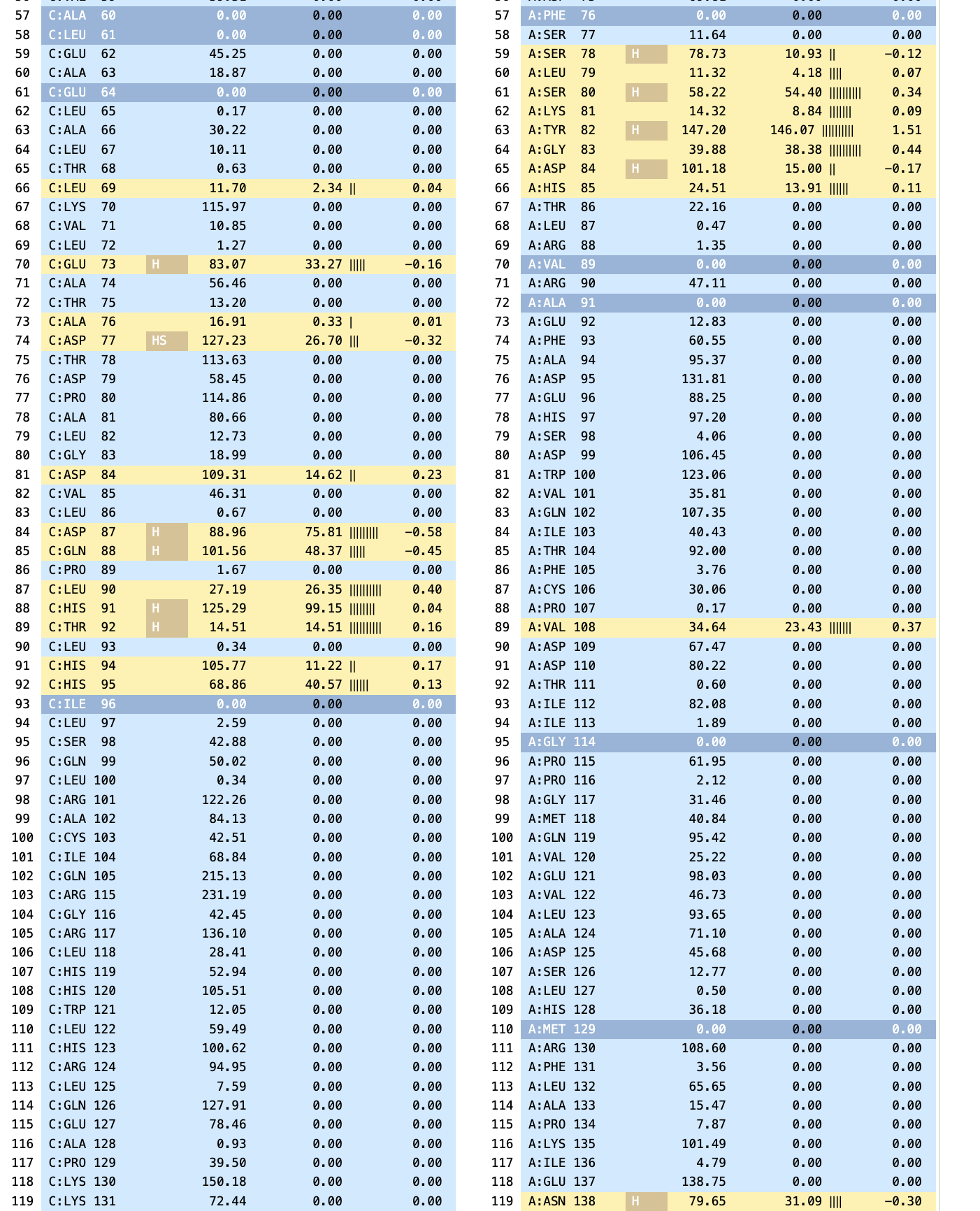
**

**
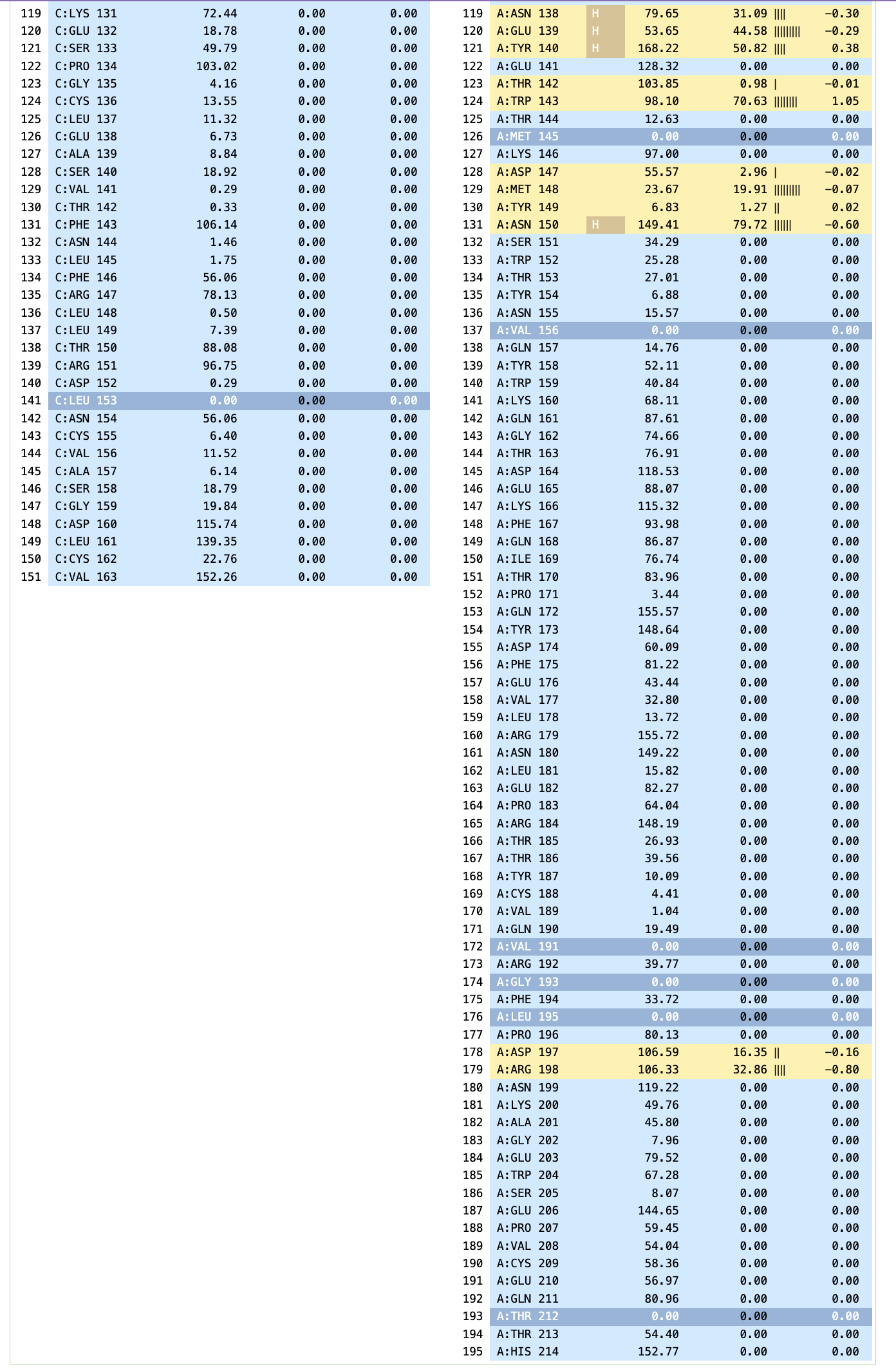
**

**Interface 2 – IFNLR1:IL10RB_A3 (B:A)**


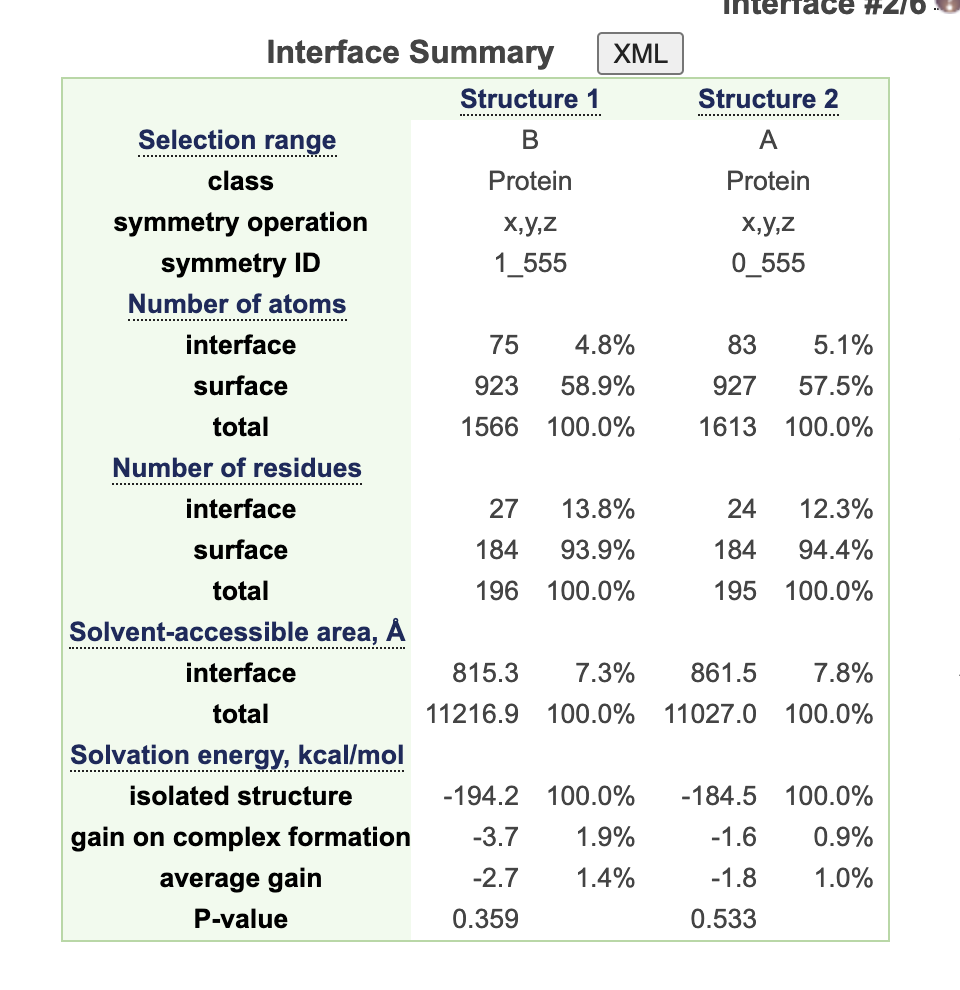


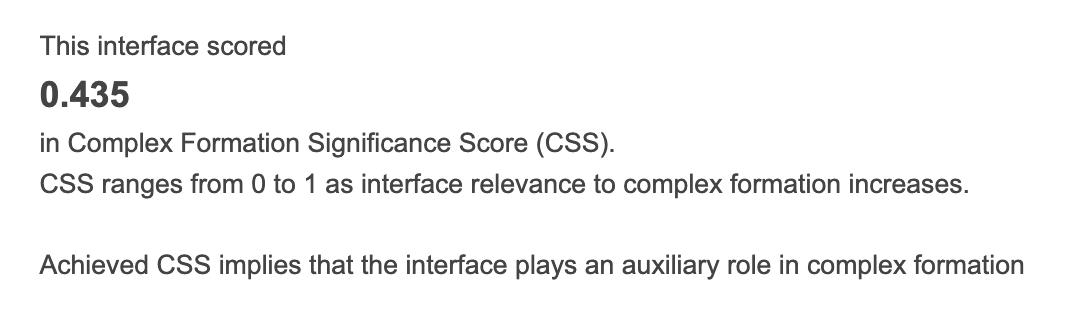


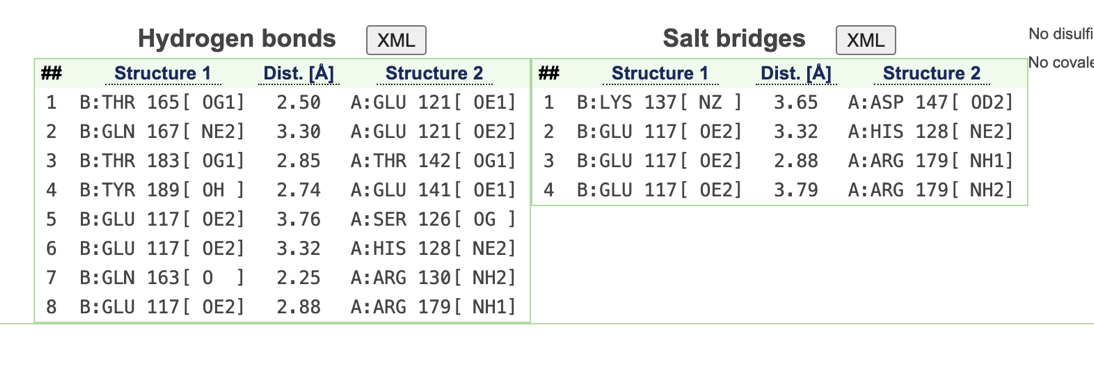


Notes:

IFNLR1: 27/196 (13.8%), IL10RB_A3: 24/195 (12.3%)

Interface scored 0.435, implying that the interface plays an auxiliary role in complex formation

IFNLR1: 815.3 A interface, 11216.9 A total (7.3%)

IL10RB_A3: 861.5 A interface, 11027 A total (7.8%)

Total surface area = 1676.8 A

12 reported interactions – 8 H-bonds and 4 salt bridges. 10 total interactions. Reported as IFNLR1:IL10RB_A3.

Glu117:Ser126 (H-bond, OE2:OG)

Glu117:His128 (H-bond, OE2:NE2) / Glu117:His128 (salt bridge, OE2:NE2)

Glu117:Arg179 (H-bond, OE2:NH1) / Glu117:Arg179 (salt bridge, OE2:NH1)

Glu117:Arg179 (salt bridge, OE2:NH2)

Lys137:Asp147 (salt bridge, NZ:OD2)

Gln163:Arg130 (H-bond, O:NH2)

Thr165:Glu121 (H-bond, OG1:OE1)

Gln167:Glu121 (H-bond, NE2:OE2)

Thr183:Thr142 (H-bond, OG1:OG1)

Tyr189:Glu141 (H-bond, OH:OE1)

Stretches of interaction –

IFNLR1: Glu116-Ile118, Asp135, Lys137, Glu139, Phe153-Pro168 (158 not included), Ile181-Tyr189 (182, 184, 188 NI)

IL10RB_A3: Gln119-Lys135 (120, 125, 127, 129, 133-134 not included), Tyr140-Asp147 (145 NI), Gln172-Arg179 (176, 178 NI)

Engineered residues –

Asp147 is at the interface and forms a salt bridge with Lys137 on IFNLR1. This is the same residue forming pairs in the IFNL4 complex, but for IFNL4 it forms two bridges with Lys137. 148 is not at this interface nor is 184.

All data –

­­­


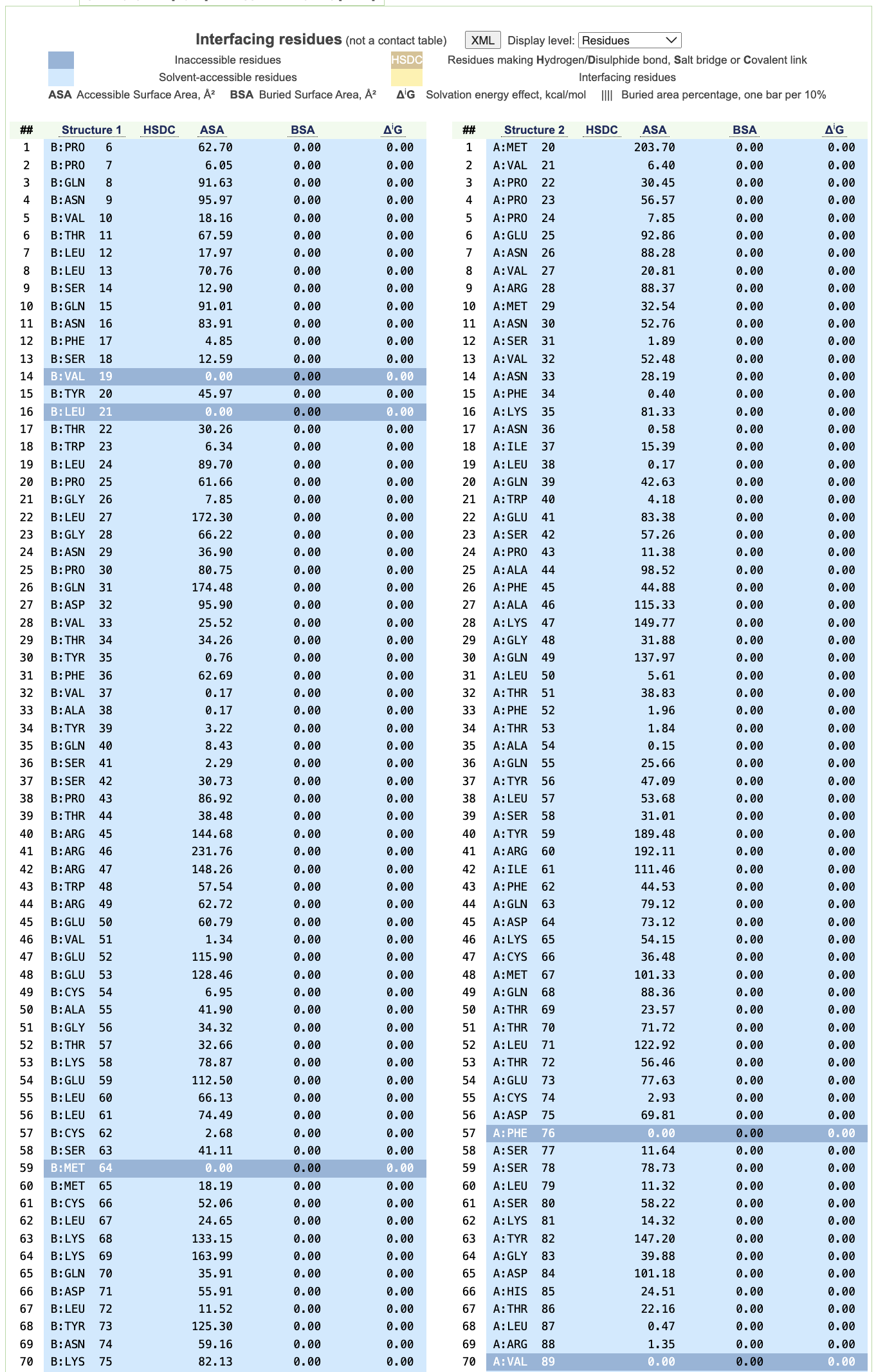


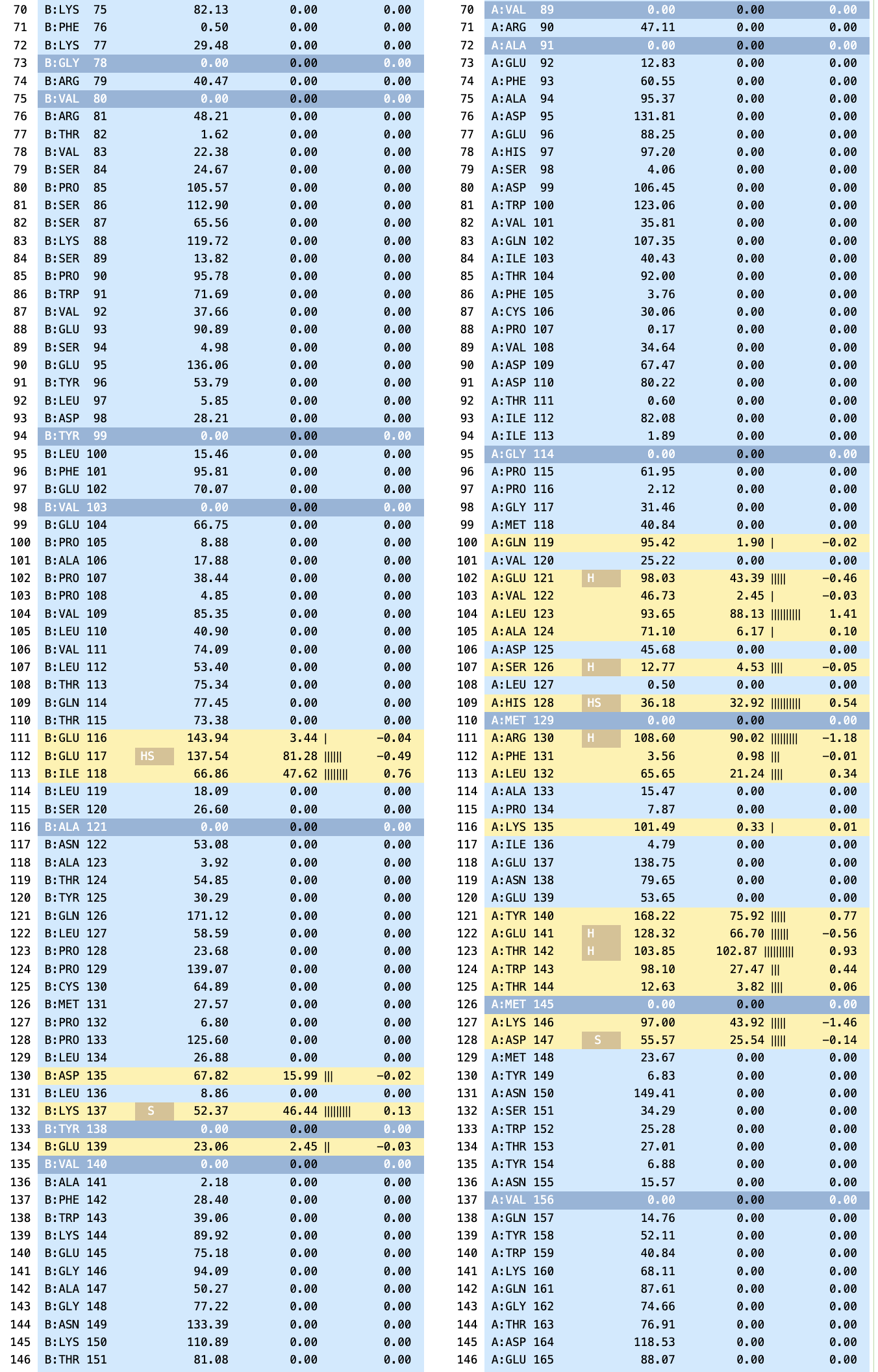


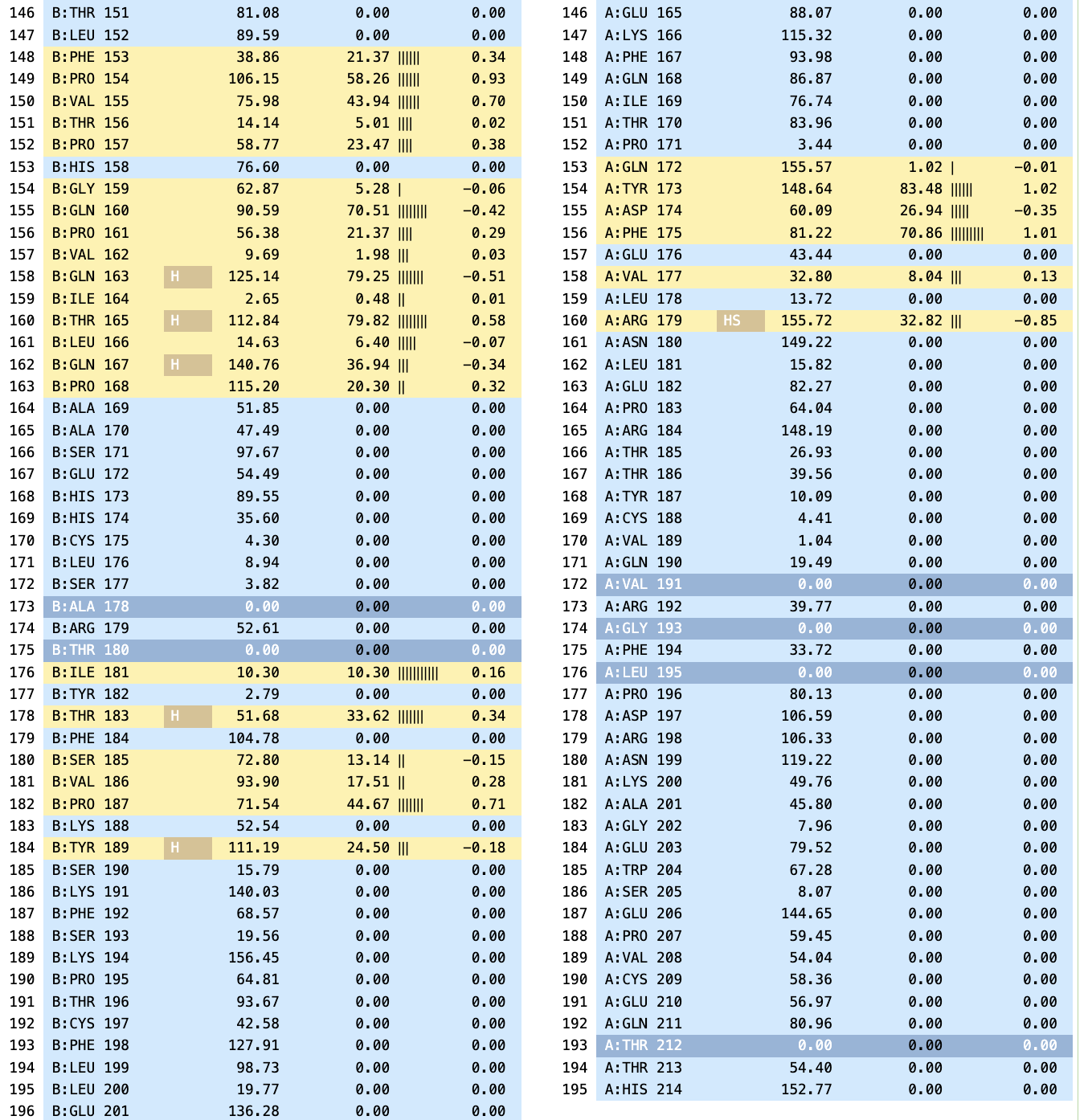


**Interface 3 – IFNL3:IFNLR1 (C:B)**


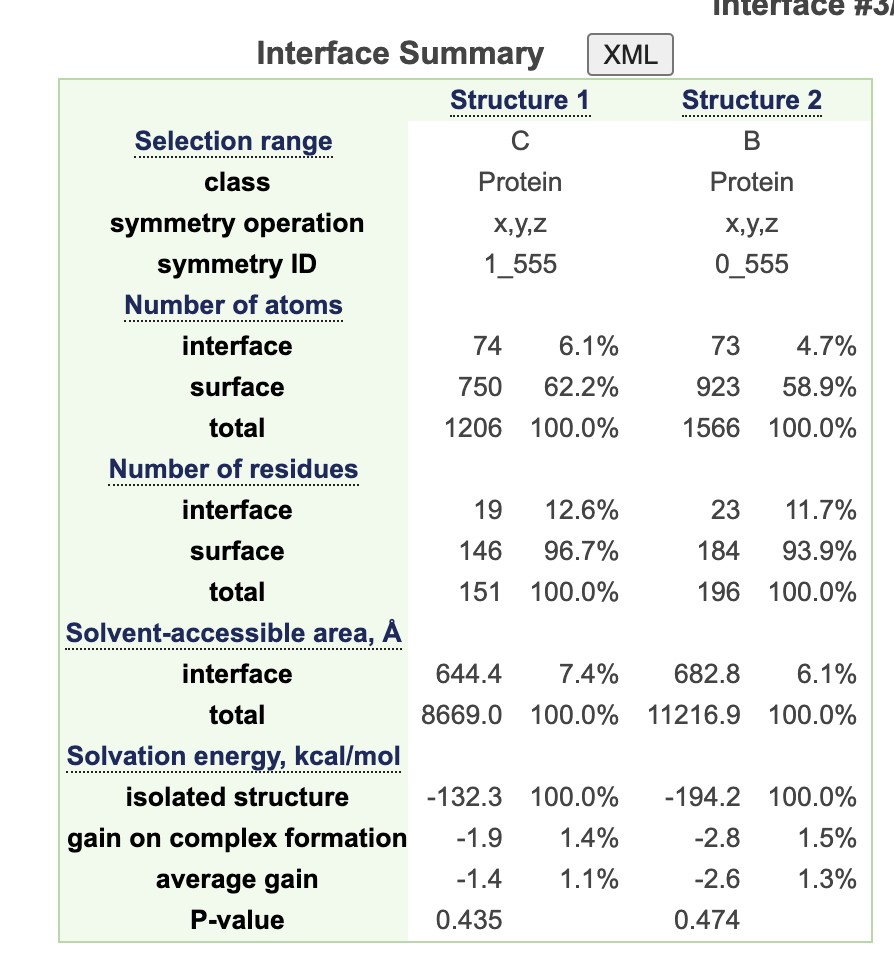


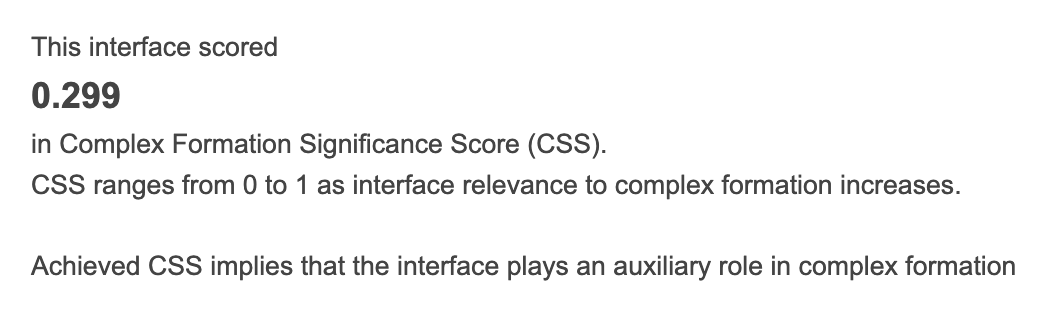


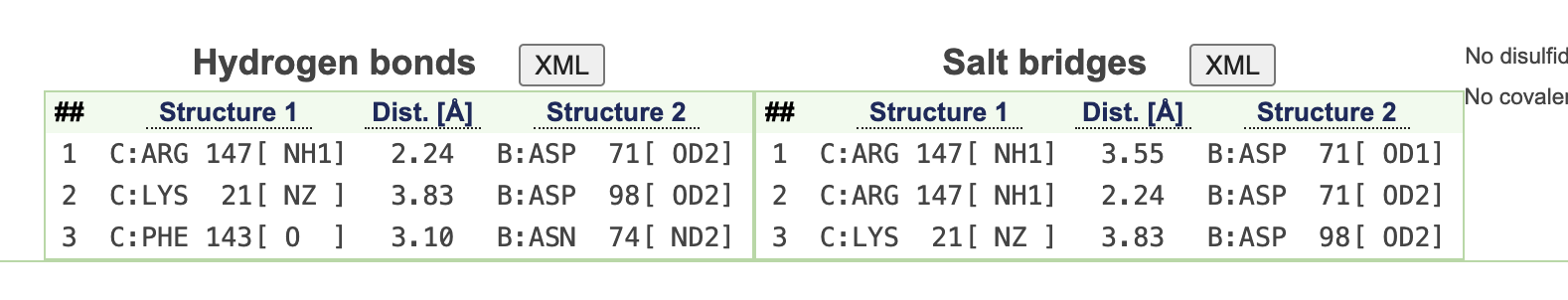


Notes:

IFNL3: 19/151 (12.6%), IFNLR1: 23/196 (11.7%)

Interface scored 0.299, implying that the interface plays an auxiliary role in complex formation

IFNL3: 644.4 A interface, 8669 A total (7.4%)

IFNLR1: 682.8 A interface, 11216.9 A total (6.1%)

Total surface area = 1327.2 A

6 reported interactions – 3 H-bonds and 3 salt bridges. 4 total interactions. Reported as IFNL3:IFNLR1.

Stretches of interaction –

IFNL3: Ser11-Leu12, Pro14, Leu17-Gln18, Lys21, Lys24-Asp25, Glu28, Leu32, Lys37, Arg41, Thr46, Phe143, Phe146-Arg147, Thr150-Arg151, Asn154

IFNLR1: Ser42-Arg49 (48 NI), Lys69-Lys75 (70, 72 NI), Asp98-Phe101 (99 NI), Pro132-Asp135 (134 NI), Thr183-Pro187

Engineered residues –

Thr150 on IFNL3 is at the interface and is very buried (~80%)

All data –

­­­


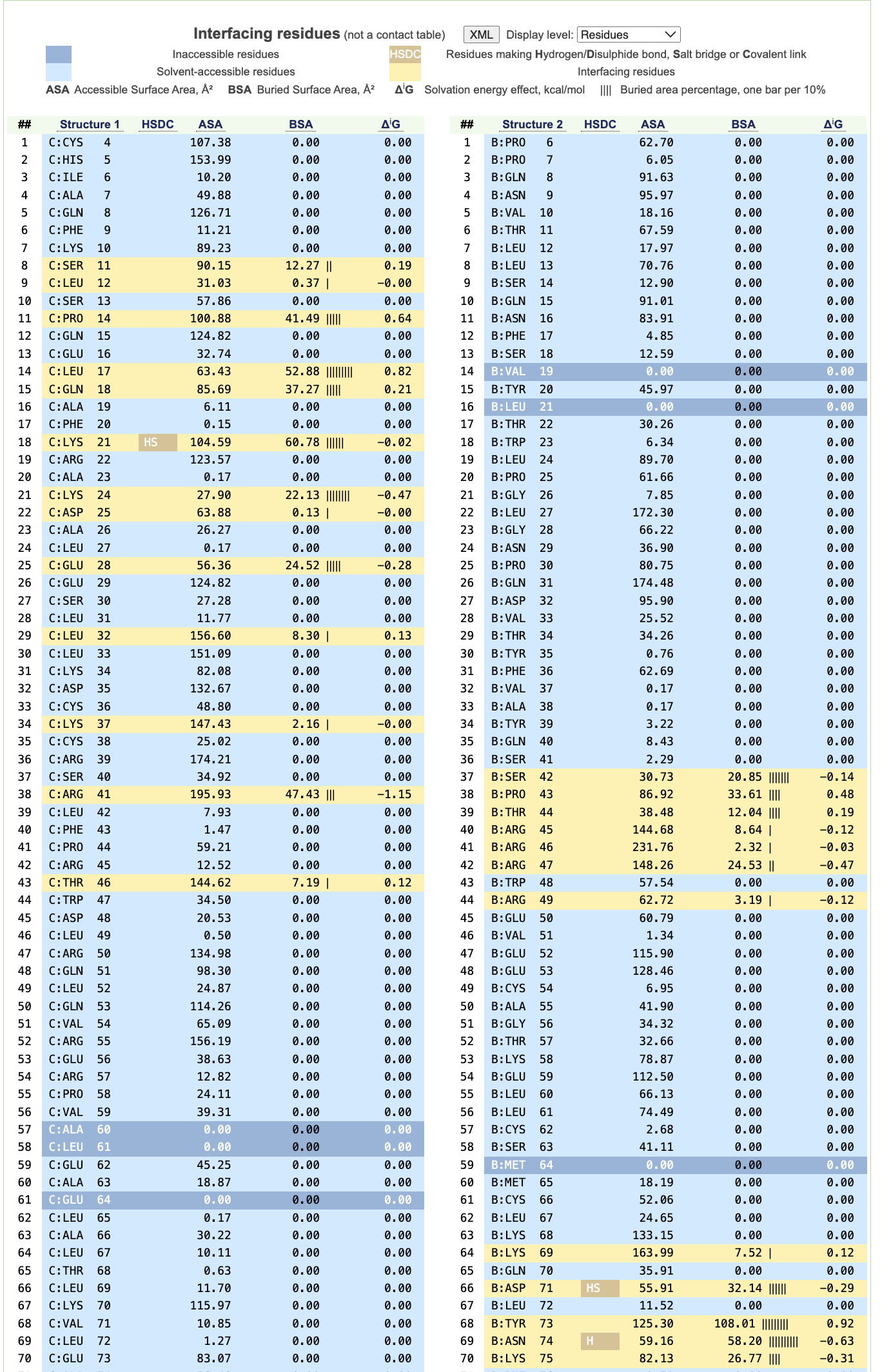


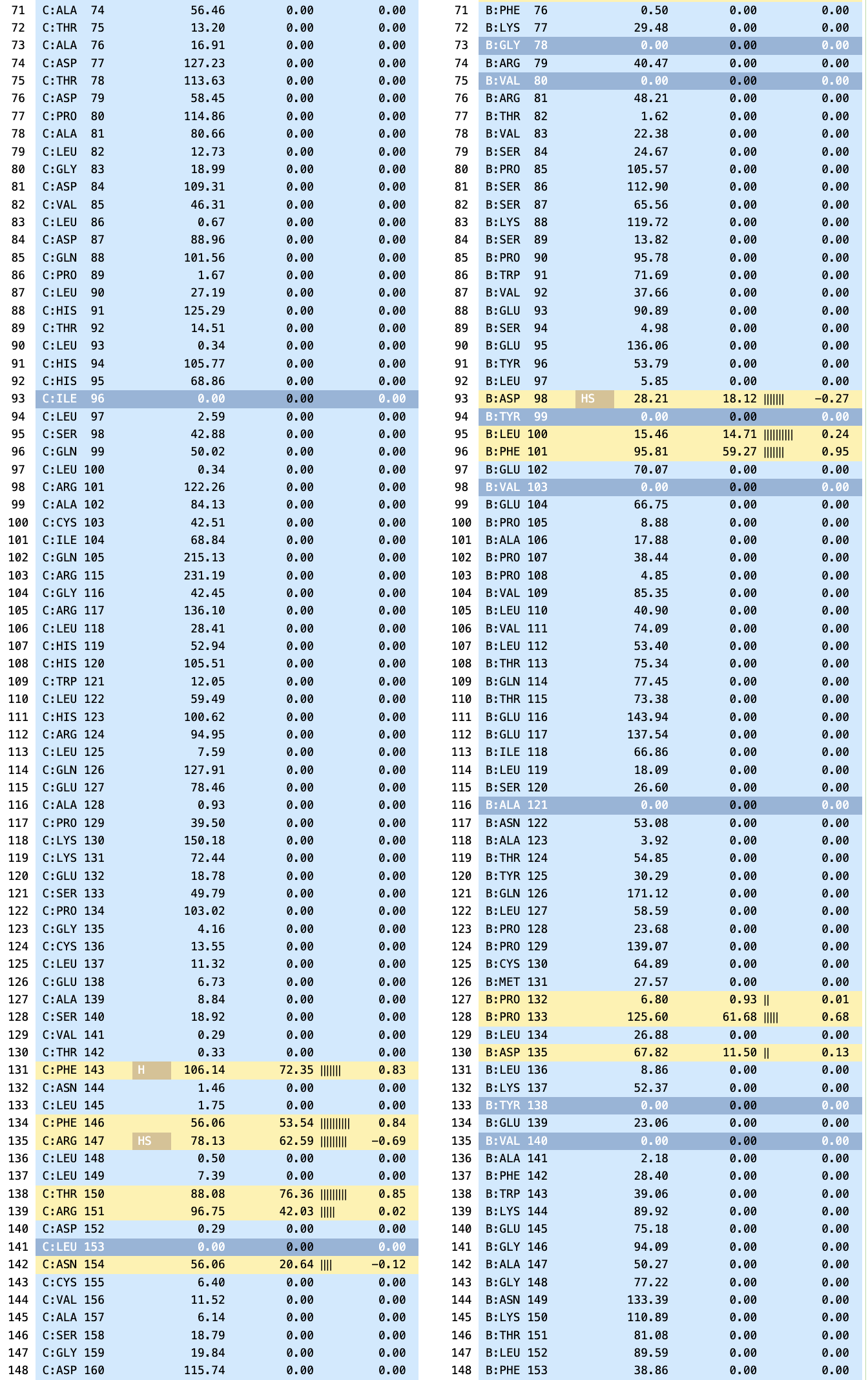


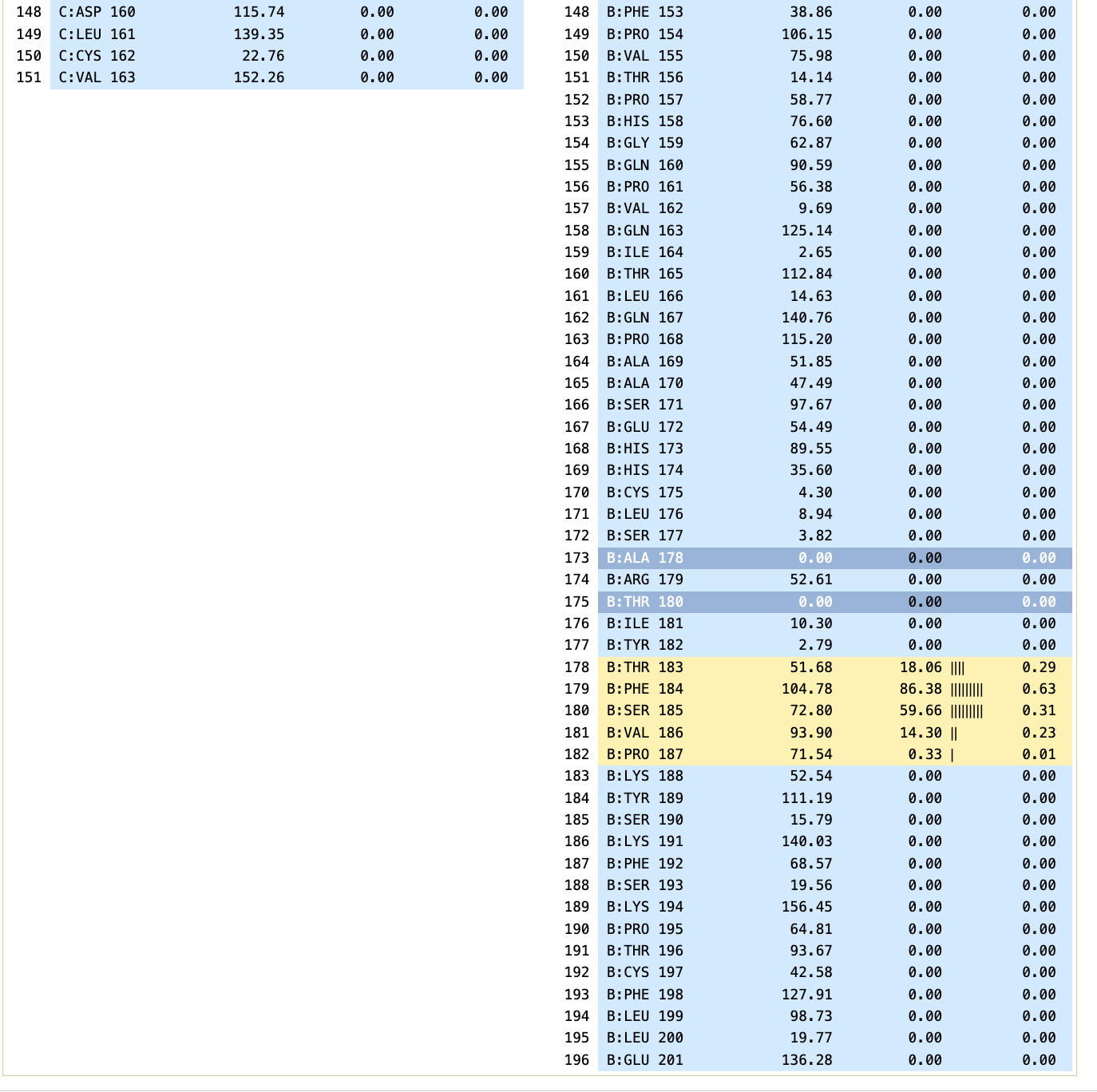

Supplement: Supplementary file 6 — Source Data [file 41467_2025_56119_MOESM6_ESM.zip › source_data/IFNL4_data/IFNL4_manuscript/Fig2/PDBePISA/interfaces/20240124_IFNL3_complex_analysis.docx]
