# Supplementary figures and images for: Structural studies of the IFNλ4 receptor complex using cryoEM enabled by protein engineering
Source: Nat Commun. 2025 Jan 18;16:818. doi: 10.1038/s41467-025-56119-y (PMC11742915; doi:10.1038/s41467-025-56119-y)

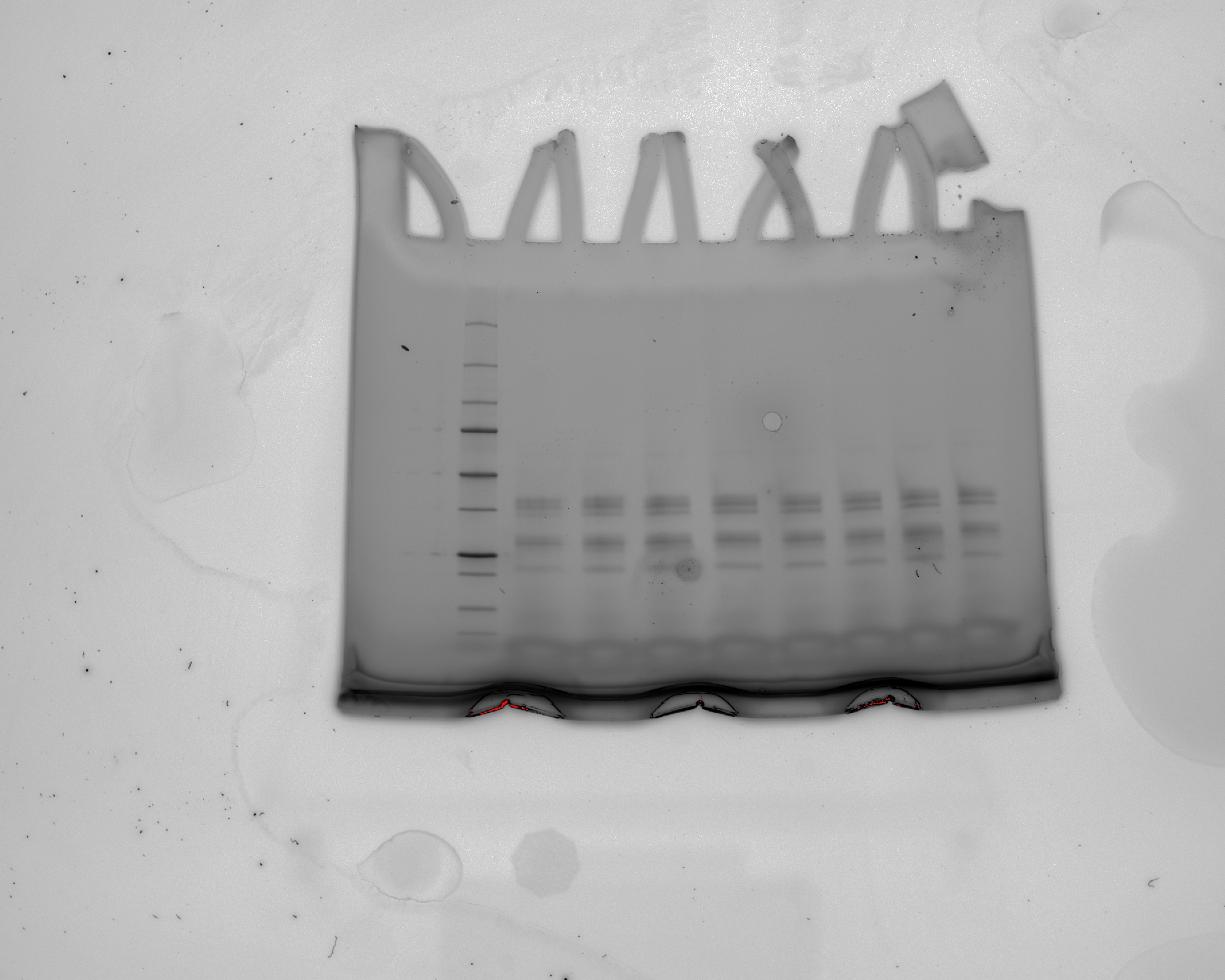

Supplement: Supplementary file 6 — Source Data [file 41467_2025_56119_MOESM6_ESM.zip › source_data/IFNL4_data/IFNL4_manuscript/Supp/SuppFig3/ifnl4_complex_gel.jpg]

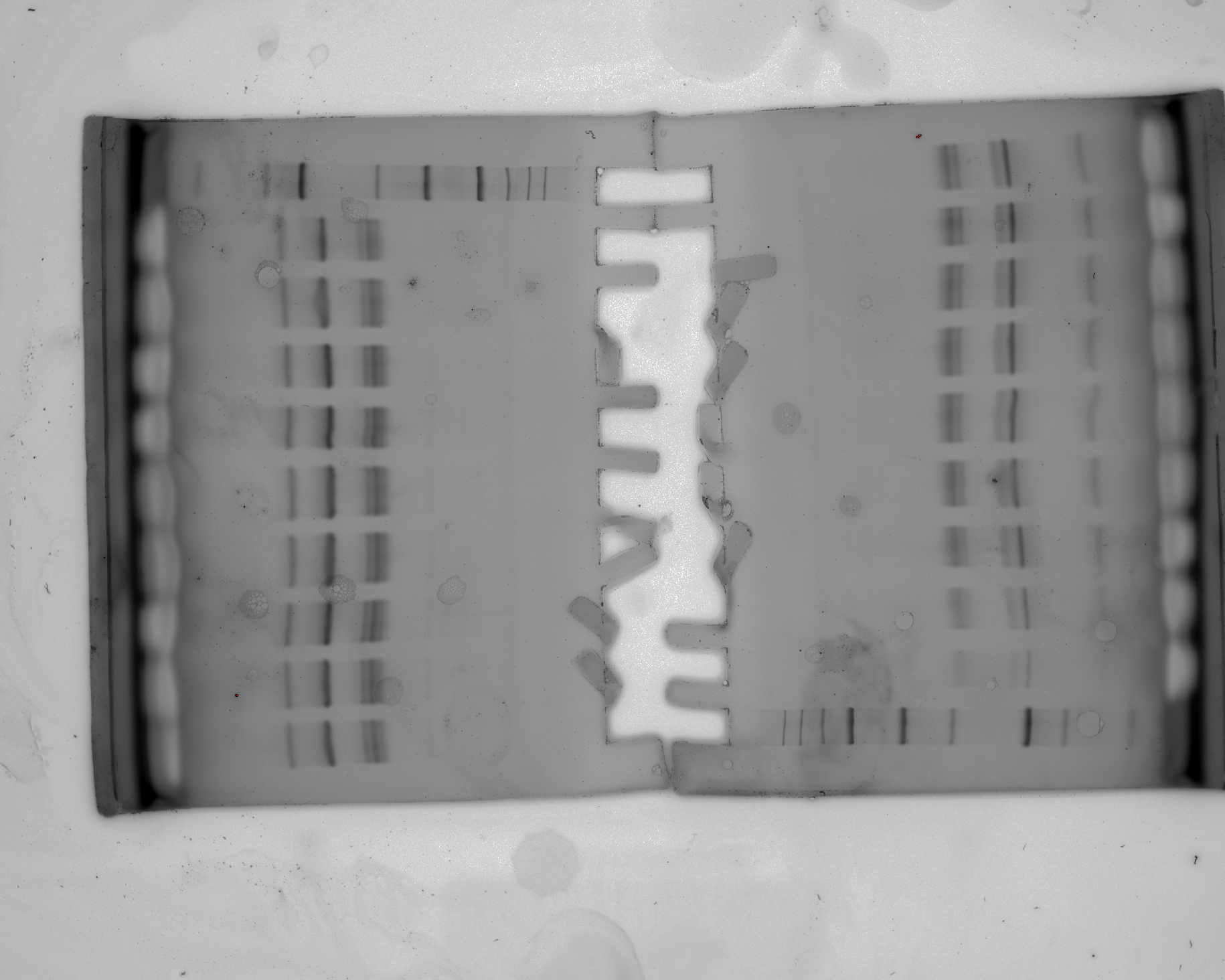

Supplement: Supplementary file 6 — Source Data [file 41467_2025_56119_MOESM6_ESM.zip › source_data/IFNL4_data/IFNL4_manuscript/Supp/SuppFig3/ifnl3_complex_gel.jpg]

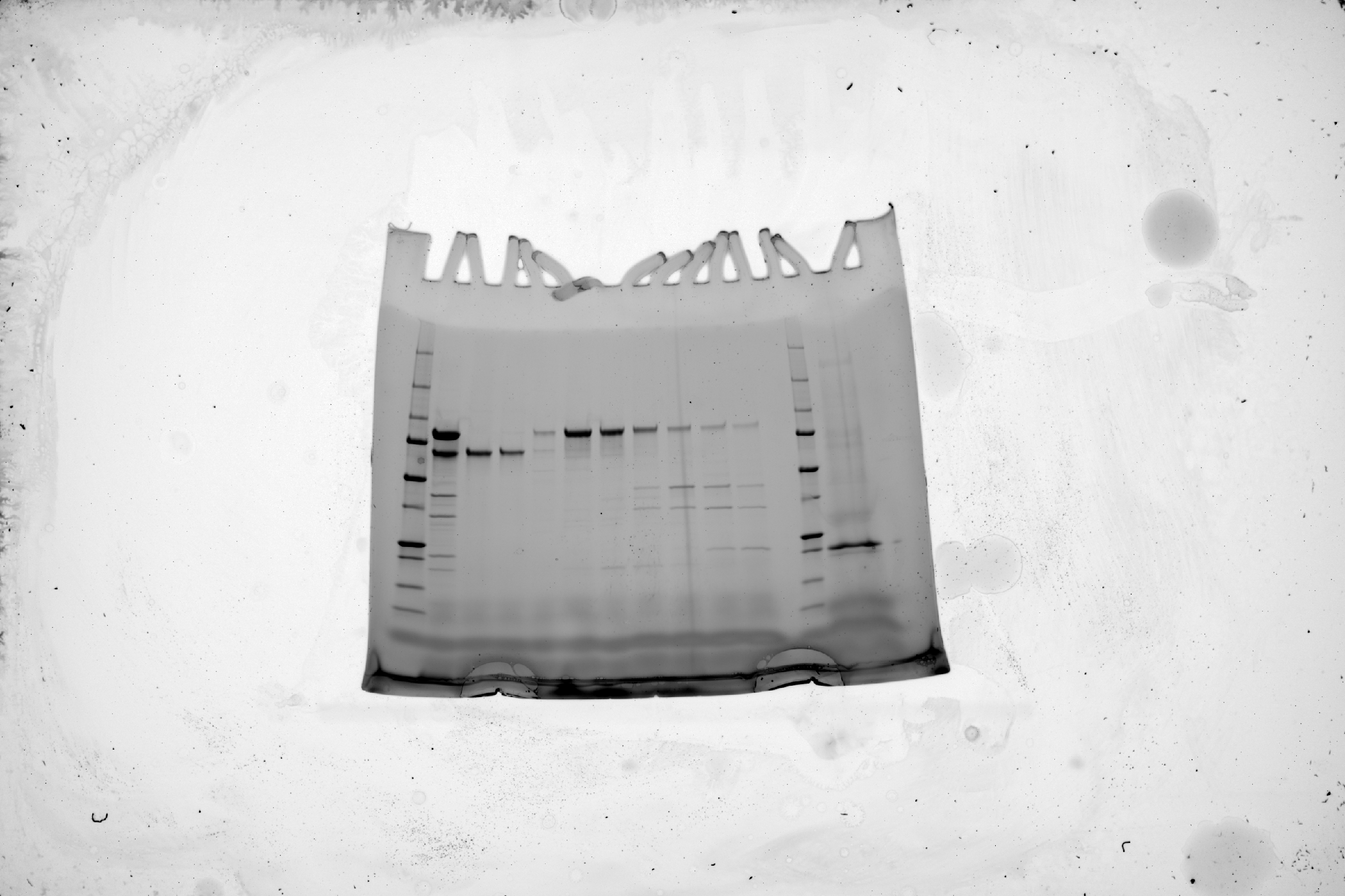

Supplement: Supplementary file 6 — Source Data [file 41467_2025_56119_MOESM6_ESM.zip › source_data/IFNL4_data/IFNL4_manuscript/Fig4/Fig4E/gel/full_gel/GelDoc Images 2024-04-19_10.53.06/full_gel_sample_lane_15.tif]
